# The control of red colour by a family of MYB transcription factors in octoploid strawberry (*Fragaria* × *ananassa*) fruits

**DOI:** 10.1111/pbi.13282

**Published:** 2019-12-12

**Authors:** Hua Wang, Hui Zhang, Yuan Yang, Maofu Li, Yuntao Zhang, Jiashen Liu, Jing Dong, Jie Li, Eugenio Butelli, Zhen Xue, Aimin Wang, Guixia Wang, Cathie Martin, Wanmei Jin

**Affiliations:** ^1^ Beijing Academy of Forestry and Pomology Sciences Beijing Academy of Agriculture and Forestry Sciences Beijing China; ^2^ Key Laboratory of Biology and Genetic Improvement of Horticultural Crops (North China) Ministry of Agriculture and Rural Affairs Beijing China; ^3^ Key Laboratory of Plant Molecular Physiology Institute of Botany Chinese Academy of Sciences Beijing China; ^4^ University of Chinese Academy of Sciences Beijing China; ^5^ Beijing Engineering Research Center for Deciduous Fruit Trees Beijing China; ^6^ John Innes Centre Norwich UK

**Keywords:** *FaMYB* genes, fruit colour, flavonoids, anthocyanins, *Fragaria* × *ananassa*

## Abstract

Octoploid strawberry (*Fragaria* × *ananassa* Duch.) is a model plant for research and one of the most important non‐climacteric fruit crops throughout the world. The associations between regulatory networks and metabolite composition were explored for one of the most critical agricultural properties in octoploid strawberry, fruit colour. Differences in the levels of flavonoids are due to the differences in the expression of structural and regulatory genes involved in flavonoid biosynthesis. The molecular mechanisms underlying differences in fruit colour were compared between red and white octoploid strawberry varieties. *FaMYB* genes had combinatorial effects in determining the red colour of fruit through the regulation of flavonoid biosynthesis in response to the increase in endogenous ABA at the final stage of fruit development. Analysis of alleles of *FaMYB10* and *FaMYB1* in red and white strawberry varieties led to the discovery of a white‐specific variant allele of *FaMYB10*, *FaMYB10‐2*. Its coding sequence possessed an ACTTATAC insertion in the genomic region encoding the C‐terminus of the protein. This insertion introduced a predicted premature termination codon, which suggested the loss of intact FaMYB10 protein playing a critical role in the loss of red colour in white octoploid strawberry*.*

## Introduction

Cultivated strawberry is a non‐climacteric fruit crop, with one of the most complicated genomes among all the crop plants. It carries eight sets of chromosomes (octoploid subspecies in genus *Fragaria*, 2*n* = 8*x* = 56) derived from four different diploid ancestors (Shulaev *et al.*, 2011). Among current cultivated strawberry subspecies, the most popular *Fragaria* subspecies (*Fragaria* × *ananassa* Duch.) was derived from the accidental crossing between the white‐fruited Chilean strawberry subspecies (*F. chiloensis* (L.) Mill.) and the red‐fruited meadow strawberry subspecies (*F. virginiana* Mill.) in the Royal Botanical Garden of the French king around 1700 (Finn *et al.*, 2013). This subspecies was subsequently named as *Fragaria* × *ananassa* by the great French botanist, Antoine Nicholas Duchesne (Hancock *et al.*, 2008). Due to the superior fruit quality of this hybrid subspecies, it was quickly distributed across the world and gradually displaced different indigenous cultivated subspecies of *F. chiloensis* in North and South America (Finn *et al.*, 2013). By the middle of the 20th century, *Fragaria* × *ananassa* and its derivatives had emerged as the most dominant cultivated strawberry varieties across the world (Hancock *et al.*, 2008). As globalization has progressed, this octoploid cultivated strawberry subspecies has grown to be one of the most important fruit crop plants and its total annual production had reached over 8.11 million tonnes by 2014 (FAO, 2014).

**Figure 1:**
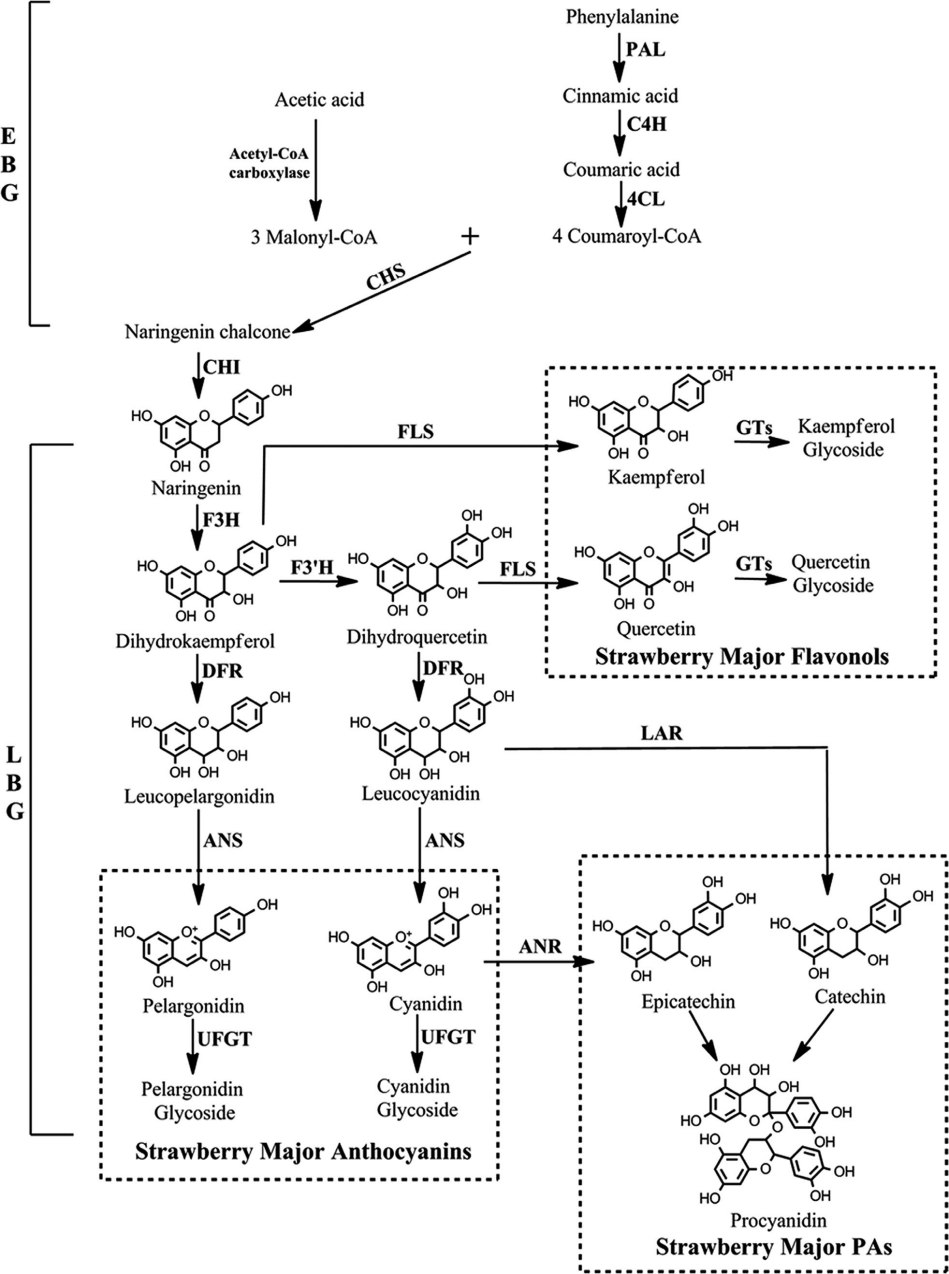
The canonical pathway of flavonoid biosynthesis in strawberry (*Fragaria* × *ananassa*) fruits. EBG, early biosynthetic genes; and LBG, late biosynthetic genes.

Horticulturists have long been interested in breeding different varieties of plants with fruits having bright colours, saturated hues, sweet taste and high nutritional value, among which attractive fruit colour is one of the most desirable horticultural traits (Kayesh *et al.*, 2013; Pillet *et al.*, 2015). The quantity and variability of flavonoids control strawberry fruit coloration in both wild diploid and cultivated octoploid strawberry varieties (Lin‐Wang *et al.*, 2014; Schaart *et al.*, 2013). Flavonoids have multiple stress‐protecting functions for plants and health‐promoting properties for humans (Allan *et al.*, 2008; Jaakola, 2013; Zhang *et al.*, 2014). Branches of the flavonoid biosynthetic pathway are involved in the production and regulation of anthocyanins, proanthocyanins and flavonols (Figure 1). Pelargonidin and cyanidin are responsible for the bright red colour and dark red colour of ripe fruits, respectively, which are also the major colours in cultivated octoploid strawberry subspecies (Fischer *et al.*, 2014). The key structural genes for flavonoid biosynthesis encode essential biosynthetic enzymes. Expression of early biosynthetic genes (EBG) (Figure 1), including phenylalanine ammonia‐lyase (*PAL*), cinnamate 4‐hydroxylase (*C4H*), 4‐coumarate:coenzyme A ligase (*4CL*), chalcone synthase (*CHS*), chalcone isomerase (*CHI*), and late biosynthetic genes (LBG) (Figure 1), including flavonol synthase (*FLS*) for the formation of flavonols, dihydroflavonol‐4‐reductase/flavanone‐4‐reductase (*DFR*), anthocyanidin synthase (*ANS*) and flavonol‐O‐glucosyltransferases (*UFGT*), is required for the formation of anthocyanins; and expression of leucoanthocyanidin reductase (*LAR*) and anthocyanidin reductase (*ANR*) is required for the formation of proanthocyanin precursors and proanthocyanins (Figure 1; Fischer *et al.*, 2014; Griesser *et al.*, 2008).

The differential accumulation of flavonoids is regulated predominantly at the transcriptional level by transcription factors (TFs) (Jin *et al.*, 2016; Lin‐Wang *et al.*, 2010). The R2R3MYB TFs are known to be major players controlling flavonoid biosynthesis during fruit coloration in many species. Another transcription factor family (bHLH) and WD40‐related proteins are also involved in the formation of a conserved ternary MBW complex together with R2R3MYB proteins. The MBW complex plays a critical role in the regulation of anthocyanin and proanthocyanin biosynthesis (Nesi *et al.*, 2000; Walker *et al.*, 1999). Furthermore, the transcript levels of key regulatory genes of flavonoid biosynthesis are tightly regulated during the process of the fruit coloration of different plants, such as *Vitis vinifera* (grapevine), *Malus domestica* (apple), *Fragaria* × *ananassa* (strawberry) and *Prunus avium* (sweet cherry; Aharoni *et al.*, 2001; Espley *et al.*, 2007; Jin *et al.*, 2016; Kobayashi *et al.*, 2002). Comparison of the closest diploid species, *F. vesca*, to octoploid *F.* × *ananassa* shows that there are 1616 gene encoding transcription factors in its genome, including 187 *MYB* and *MYB*‐related transcription factors (Shulaev *et al.*, 2011). Several recent studies have demonstrated that how several R2R3MYB family members participate in the regulation of branches of flavonoid biosynthesis in the cultivated strawberry variety (*F*. × *ananassa*), indicating that FaMYB1 (Paolocci *et al.*, 2011), FaMYB9/FaMYB11 (Schaart *et al.*, 2013), FaMYB5 (Schaart *et al.*, 2013) and FaMYB10 (Kadomura‐Ishikawa *et al.*, 2015; Lin‐Wang *et al.*, 2010) are key TFs involved in regulating flavonoid biosynthesis in response to hormones, abiotic stress, biotic stress, light and circadian rhythms. However, little is known about the regulatory network controlling fruit colour in octoploid strawberry varieties.

To broaden this understanding, ‘Snow Princess’, a white octoploid strawberry variety (WS), and ‘Sweet Charlie’, the red octoploid strawberry (RS) variety, were subjected to qualitative and quantitative LC‐MS/MS analyses of flavonoid composition, co‐expression analysis of regulatory and structural genes related to flavonoid biosynthesis, and sequence analyses of two key TFs for anthocyanin biosynthesis, *FaMYB10* and *FaMYB1*. Based on the homology of R2R3 domains of the MYB transcription factors from different plant species, as well as the red and white strawberry varieties, phylogenetic analysis demonstrated that FaMYB1, FaMYB9, FaMYB10 and FaMYB11 were closely related, structurally to the MYB proteins with known functions in regulating flavonoid metabolism in other species (Dubos *et al.*, 2010; Shulaev *et al.*, 2011). The regulatory network of strawberry fruit coloration was studied using these TFs in combination with the changes in expression of key flavonoid structural genes and the levels of endogenous ABA by principal component analysis. A working model is proposed to explain the combinatorial effects of *FaMYB1*, *FaMYB10; FaMYB9* and *FaMYB11* in regulating different branches of flavonoid biosynthesis in ripe fruit.

Allele is one of two or more alternative forms of a gene at the same site in a chromosome, which determine alternative characters that can be passed on from parents to offspring through sexual reproduction. The cultivated strawberry has four subgenomes, each locus can represent up to different alleles in a single individual (Lin‐Wang *et al.*, 2010; Lin‐Wang *et al.*, 2014). Analyses of *FaMYB10* and *FaMYB1* alleles in WS revealed that *FaMYB10‐2,* a WS‐specific allele of *FaMYB10*, contains an insertion (ACTTATAC) resulting in a presumed premature stop codon in the *FaMYB10* coding sequence. Interestingly, the genomic sequence of *FaMYB10‐2* was intronless, compared to the RS‐specific allele *FaMYB10‐1*. These structural changes in the *FaMYB10‐2* allele are likely to be the primary reasons for the striking colour difference between RS and WS due to the loss of the *FaMYB10*‐mediated up‐regulation of anthocyanin biosynthesis in ripe fruit. *FaMYB1* alleles showed only minor differences between RS and WS varieties.

## Results

### Analyses of changes in flavonoid composition and endogenous ABA levels during strawberry fruit ripening

**Figure 2:**
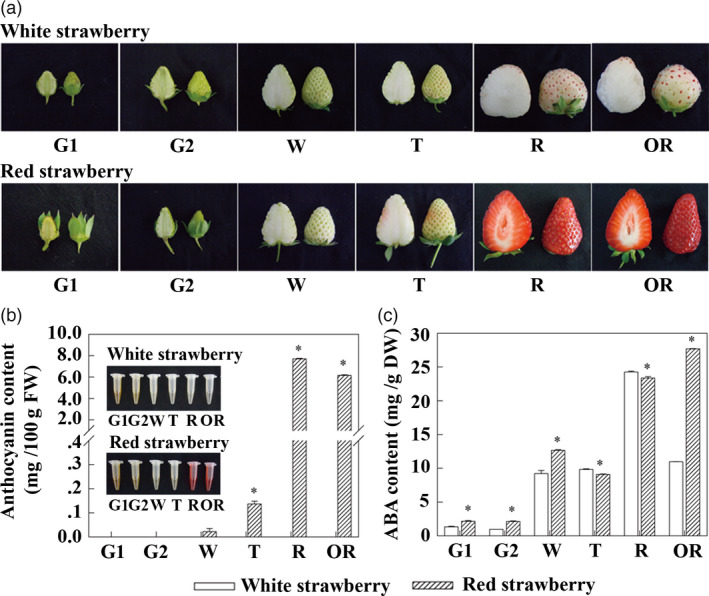
Changes in the anthocyanin content and in endogenous ABA levels during strawberry fruit ripening. (a) The strawberry fruits at the different visual fruit developmental stages used in this study. (b) The anthocyanin content in white and red strawberry varieties. (c) The endogenous ABA levels in white and red strawberry varieties. Asterisks (*) represent that the values of total anthocyanin content (*n* = 3, ±SE) are significantly different at *P* < 0.05 as determined using independent *t*‐test.

Six visual distinguishable developmental stages of strawberry fruits were defined; small green (G1), large green (G2), white (W), turning (T), ripe (R) and over‐ripe (OR), which were minor modifications of stages defined in previous reports (Moyano *et al.*, 1998; Schaart *et al.*, 2013). For WS, these stages were defined by seed colour examined in the field (see Methods; Figure S1) at 13, 17, 24, 30, 46 and 50 days after anthesis (Figure 2a, top), and for RS, these stages were defined by fruit colour at 7, 12, 17, 19, 26 and 30 days after anthesis (Figure 2a, bottom). The coloration of fruit varied significantly between WS and RS (Figure 2a). Fruit coloration is greatly influenced by the differential accumulation of anthocyanins; therefore, anthocyanin contents during fruit development were assessed. In WS, anthocyanins were almost undetectable at any of the six developmental stages; in RS, anthocyanins were undetectable during G1, G2 and W stages, but increased rapidly from 0.13 mg/100 g FW at the T stage to 7.70 mg/100 g FW at the R stage (Figure 2b). Analysis using LC‐MS/MS showed that the major anthocyanin was pelargonidin‐3‐glucoside (Table 1; Figure S2). The concentration of total anthocyanins in WS fruits was 42.43% at the T stage and 0.15% at the R stage (Table S1) of those in RS correspondingly.

**Table 1:** Levels of anthocyanidins, flavonols and proanthocyanins in red and white strawberry varieties

Peak No.	Tentative identification	White strawberry turning phase (μg/g)	Red strawberry turning phase (μg/g)	White strawberry ripe phase (μg/g)	Red strawberry ripe phase (μg/g)	Cat.
1	Procyanidin B1	819.07 ± 57.61^b^	1615.64 ± 103.92^c^	329.91 ± 32.55^a^	507.02 ± 28.67^a^	PA
2	Proanthocyanidins	765.69 ± 44.69^ab^	507.86 ± 42.69^a^	592.92 ± 66.43^a^	1209.62 ± 189.52^b^	PA
3	Cyanidin‐3,5‐diglucoside	4.52 × 10^‐3^ ± 0.00^a^	5.67 × 10^‐3^ ± 0.00^a^	5.11 × 10^‐3^ ± 0.00^a^	0.01 ± 0.00^b^	AC
4	Pelargonidin‐3‐glucoside	189.89 ± 6.07^a^	447.11 ± 7.38^a^	188.46 ± 3.35^a^	125389.52 ± 8702.50^b^	AC
5	Pelargonidin 3‐acetyl‐glucoside	0.03 ± 0.01^a^	0.54 ± 0.04^a^	N/A	1029.32 ± 63.37^b^	AC
6	Kaempferol‐3‐glucoside	16.35 ± 1.06^a^	228.94 ± 24.77^b^	6.14 ± 0.33^a^	36.38 ± 2.38^a^	FL
7	Kaempfeol‐3‐rutinoside	1.34 ± 0.22^b^	5.96 × 10^‐3^ ± 0.00^a^	5.59 ± 0.36^c^	N/A	FL
8	Quercetin 3‐glucoside	7.78 ± 1.17^b^	31.33 ± 1.06^d^	1.77 ± 0.30^a^	15.91 ± 1.20^c^	FL
9	(+/‐)‐Catechin	1608.66 ± 112.13^c^	977.59 ± 32.54^b^	161.21 ± 24.11^a^	204.30 ± 12.81^a^	PP
10	Epicatechin	14.66 ± 0.48^b^	6.12 ± 0.57^a^	5.73 ± 0.58^a^	5.21 ± 0.43^a^	PP

Values represent mean ± SE. a, b, c, d letters indicate statistically significant differences within levels of anthocyanidins, flavonols and proanthocyanins in red and white strawberry varieties (Duncan's honestly significant difference test, *p* < 0.05).

AC, anthocyanins; FL, flavonols; PA, proanthocyanins; PP, precursors of proanthocyanidins, catechin, and epicatechin.

Proanthocyanins were detected in both RS and WS fruit. The major proanthocyanin was procyanidin B1, and other proanthocyanidins were detected as well (Table 1). In WS, the content of total proanthocyanins was 74.63% at T stage, and 53.76% at R stage, of the values in the red strawberry variety although the major precursors of proanthocyanins accumulated in WS fruit to 165.02% of the levels in RS early during strawberry fruit development (T stage) (Table S1). Flavonols in strawberry fruit contain a major component, Pelargonidin‐3‐glucoside, and a minor component, quercetin‐3‐glucoside (Table 1). The concentration of total flavonols in WS was 9.79% of that in RS fruit at the T stage and 25.82% at the R stage. However, the total amount of flavonols in fruit of both varieties was much lower than either anthocyanins or proanthocyanins, especially at the R stage (Table S1), suggesting that the flavonol biosynthesis was suppressed in ripe fruit. Among the three flavonoids, there were 10 times more anthocyanins than the other flavonoids at the R stage.

Exogenous application of ABA has been reported to affect the accumulation of anthocyanins in strawberry through the action of *FaMYB* class genes (Ayub *et al.*, 2016; Kadomura‐Ishikawa *et al.*, 2015), so the concentration of endogenous ABA was examined in fruit throughout the fruit developmental stages. There were significant differences in ABA levels at G1, G2 and W stages with slightly higher levels of endogenous ABA in RS compared to WS. Endogenous ABA showed slightly higher levels at the critical stages of fruit coloration, the T and R stages, in WS, but reached its highest level at the OR stage in RS, and at this stage, the endogenous ABA level was significantly higher in RS than WS (Figure 2c). Based on the changes in endogenous ABA levels and flavonoids, the colour difference between WS and RS was most closely associated with the anthocyanin contents at the T and R/OR developmental stages. Therefore, our analyses of molecular mechanisms controlling colour in fruit focused on the T and R stages.

### Expression analyses of key genes of flavonoid biosynthesis

**Figure 3:**
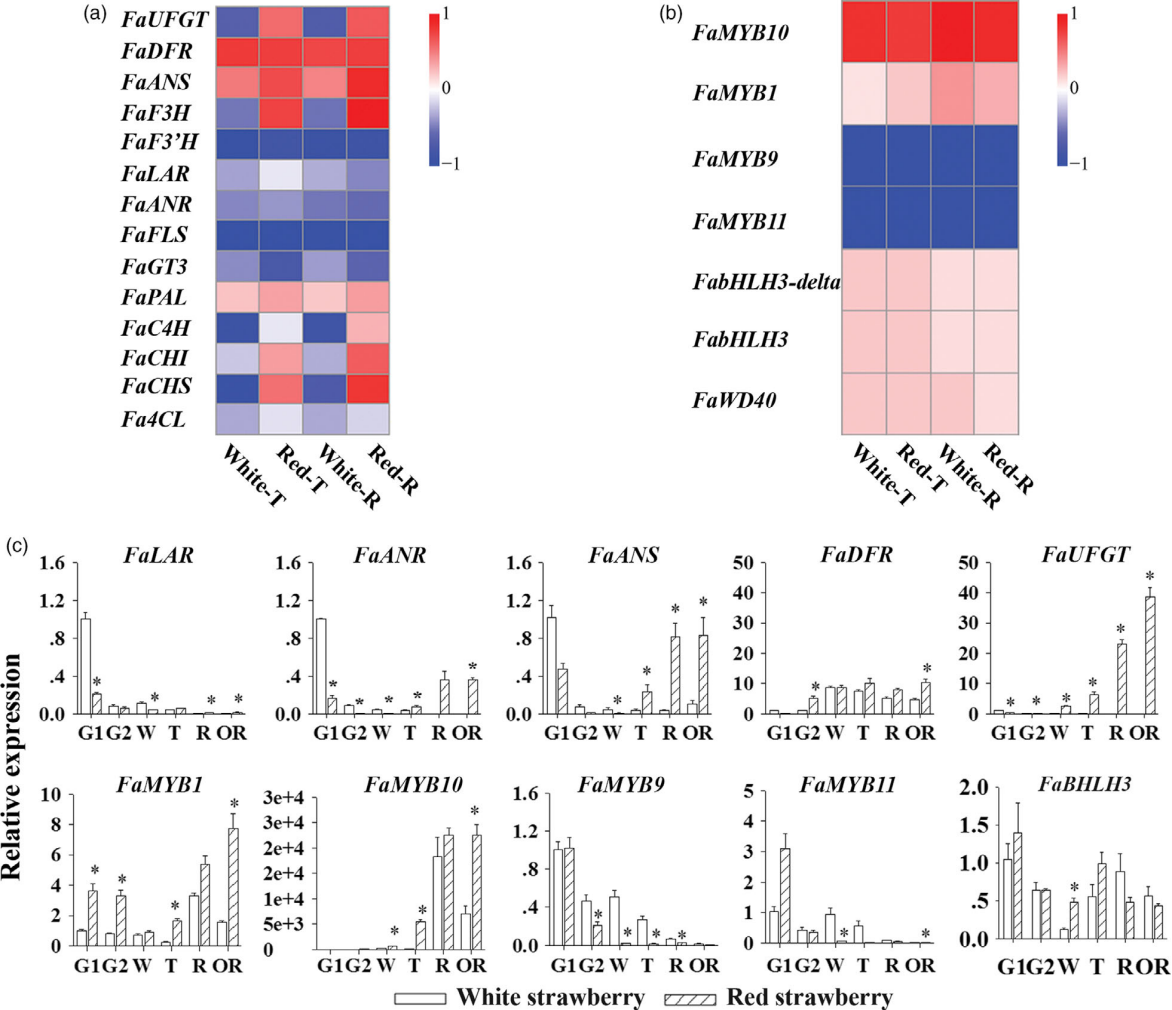
Expression analyses of key genes of flavonoid biosynthesis. (a) RNA‐seq heatmap analysis for transcript levels of structural genes in the white and red strawberry varieties. (b) RNA‐seq heatmap analysis for transcript levels of regulatory genes in the white and red strawberry varieties. (c) The real‐time PCR results for transcript levels of structural genes and regulatory genes in the white and red strawberry varieties. Asterisks (*) represent that the values of the corresponding transcription levels (*n* = 3, ±SE) are significantly different at *p* < 0.05 as determined using independent *t*‐test.

Many studies have shown that most structural genes in flavonoid biosynthesis are regulated at the transcriptional level by MYB class transcription factors. RNA‐seq analyses were performed for WS and RS fruit at the T and R developmental stages. Several key structural and regulatory genes involved flavonoid biosynthesis were selected for further analysis using standardized transcript levels, shown as heatmaps (Figure 3a and b). These genes included the structural genes *FaPAL*, *FaCHI*, *FaCHS*, *FaC4H*, *Fa4CL*, *FaFLS*, *FaF3' H*, *FaGT3*, *FaANR*, *FaLAR*, *FaF3H*, *FaDFR*, *FaANS* and *FaUFGT* (Figure 3a) and the key regulatory genes *FaMYB1*, *FaMYB10*, *FaMYB9*, *FaMYB11*, *FabHLH3*, *FabHLH3‐delta* and *FaWD40* (Figure 3b). A group of EBG genes (*FaCHI*, *FaCHS*, *FaC4H*, and *Fa4CL*) showed significantly higher expression levels in RS compared to WS at T and R stages, except for the gene, *FaPAL*, encoding the first committed enzyme of general phenylpropanoid metabolism (Figure 3a). The likely reason that collectively FaPAL transcripts showed little relative difference in transcript levels between WS and RS is that PAL is encoded by multiple genes with differential expression, and while their overall transcript levels may not differ by much, selected isoforms may be differentially expressed in WS and RS fruit. Several structural genes (*FaFLS*, *FaGT3*, *FaANR*, and *FaLAR*) had low transcript levels in both WS and RS (Figure 3a). Significantly, there was a sharp increase in transcript levels of *FaF3H*, *FaDFR*, *FaANS*, and *FaUFGT* in RS at T and R stages, which were directly correlated with anthocyanin production. And expression levels of these genes did not increase in WS fruit (Figure 3a).

To confirm these differences, the transcript abundance of the structural genes was analysed by qRT‐PCR throughout the six developmental stages of strawberry fruits. Transcript levels were standardized to the *Actin* gene and shown as bar graphs (Figure 3c, top; Figure S3). The expression levels of key genes *FaPAL*, *FaC4H*, *Fa4CL*, *FaCHS* and *FaCHI*, which are responsible for the synthesis of common precursors of flavonoid biosynthesis, were all significantly higher in RS compared to WS, similar to the results of the RNA‐seq analysis (Figure S3). Consistent with the large difference in anthocyanin contents between WS and RS from biochemical assays, the key structural genes, *FaANS*, *FaDFR* and *FaUFGT* (Figure 1) showed the biggest differences in expression levels between the two varieties (Figure 3c, top). In RS, these key anthocyanin biosynthetic genes showed a gradual increase in transcript levels until R and/or OR stages (Figure 3c, top; Table S2). In WS, transcripts of these genes peaked at lower levels during R and/or OR stages (Figure 3c, top). *FaUFGT*, encoding the last enzyme for anthocyanin biosynthesis, showed the most significant differences between RS and WS, which was 5572.49 times higher in RS relative to WS. As genes involved in proanthocyanin biosynthesis, *FaANR* and *FaLAR* showed an early peak at the G1 stage in both strawberry varieties (Figure 3c, top). Both genes exhibited much lower expression levels at G1 stage in RS (Figure 3c; Table S3) compared to WS, in accordance with the significantly higher amounts of both proanthocyanin precursors and proanthocyanins in WS fruit at this stage. *FaFLS*, which encodes flavonol synthase for flavonol biosynthesis, peaked in transcript levels at the early G2 stage in RS and at W and T stages in WS, and remained at a very low level through fruit maturation stages R and OR in both strawberry varieties, confirming our findings on the low levels of flavonols in mature fruit.

*FaANS* encodes anthocyanidin synthase and is active as a common biosynthetic step for both anthocyanin and proanthocyanin biosyntheses (Figure 1). *FaANS* showed high transcript levels at the G1 stage in WS (Figure 3c; Table S2), which was 2 times higher than that in RS at the G1 stage (Table S2), supporting the role of ANS in proanthocyanin biosynthesis. In summary, different branches of flavonoid biosynthesis showed differential transcript levels of key structural genes between RS and WS; the genes responsible for anthocyanin biosynthesis showed the greatest differences between RS and WS. Proanthocyanin biosynthesis is also different in WS compared to RS. The fact that transcript levels of all anthocyanin biosynthetic genes were much lower in ripe fruit of WS than of RS suggested that it was a difference in the regulation of expression of the structural genes encoding enzymes of anthocyanin biosynthesis that gave rise to the differences in colour between WS and RS.

Among the genes encoding FaMYB transcription factors, relative expression levels of *FaMYB1*, *FaMYB10*, *FaMYB9* and *FaMYB11*, and related regulatory genes, *FabHLH3*, *FabHLH3‐delta* and *FaWD40*, were compared in WS and RS at T and R stages (Figure 3b). Two distinct patterns of transcript levels between RS and WS were observed for the *FaMYB* genes. One showed significantly higher transcript levels in RS compared to WS, including *FaMYB1* and *FaMYB10* (Figure 3b). The other showed significant but much smaller increases in WS compared to RS and included *FaMYB9* and *FaMYB11* (Figure 3b). Furthermore, most of the genes encoding TFs present in the MBW complex together with FaMYB proteins showed similar transcript levels at T and R stages in RS except for *FabHLH3‐delta* (Figure 3b). In fact, *FabHLH3‐delta* has been suggested to be a non‐functional gene lost from the complex regulating the anthocyanin biosynthetic pathway (Schaart *et al.*, 2013).

The expression levels of the *FaMYB* genes were analysed by qRT‐PCR throughout the development of strawberry fruits and were calculated relative to the *Actin* gene as bar graphs, including the *FaMYB* genes and one *FaMYB*‐related gene, *FaWD40* (Figure 3c, bottom; Figure S4). The expression level of *FaMYB1* decreased gradually from G1 to T stage in both RS and WS, but started to rise again from the T stage and reached a peak level at the R or the OR stages, respectively (Figure 3c, bottom; Table S4). In RS, there was an early peak of *FaMYB1* transcripts at the G1 and G2 stages (Table S4). The transcript levels of *FaMYB10* were maintained at a low level from G1 to W but showed a rapid increase from the T stage to a peak level at the R stage in both WS and RS (Table S4). Thereafter, *FaMYB10* transcript levels were maintained at peak levels until the OR stage but only in RS (Figure 3c; Table S4). The expression levels of *FaMYB9* and *FaMYB11* decreased rapidly throughout the development of strawberry fruits (Figure 3c, bottom) from their peak levels at the G1 stage (Table S5), consistent with the expression patterns of their putative target structural genes, *LAR* and *ANR*. Analysis of the related genes, *FaWD40* and *FabHLH3*, showed high levels of transcripts at the G2 stage in both RS and WS (Figure 3c; Table S4), and remained at similar levels at the R and OR stages (Table S4), suggesting that the significant differences in anthocyanin contents, between RS and WS, were not due to differences in transcript levels of these genes. The peak level of *FaMYB10* expression was much higher than that of *FaMYB1* (Figure 3c, bottom), and *FaMYB10* also had a higher expression level at G1 than *FaMYB9* and *FaMYB11* (Table S5), suggesting that *FaMYB10* might be involved in both proanthocyanin and flavonol biosyntheses during the early stages of fruit development. Our data suggested that *FaMYB10* plays a prominent role in creating the colour difference between RS and WS.

### Coding sequences of *FaMYB10* are disrupted in WS, potentially contributing to a loss of function allele

**Figure 4:**
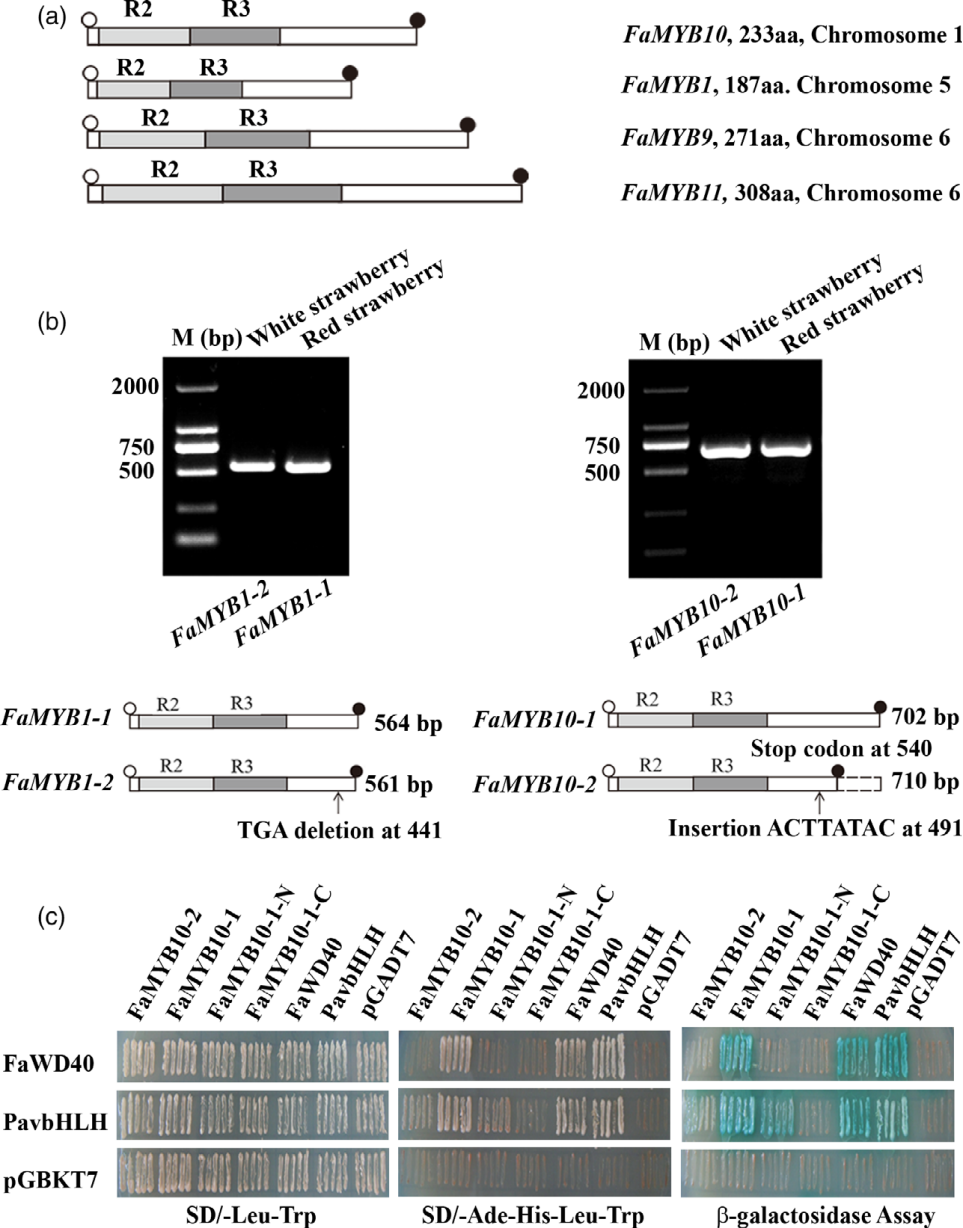
Characterization of *FaMYB1* and *FaMYB10* coding sequences in the white and red strawberry varieties. (a) Coding domain structures of *FaMYB1*, *FaMYB10*, *FaMYB9*, *FaMYB11*. (b) The agarose gel of *FaMYB1* and *FaMYB10* alleles and the missing sequence in *MYB10‐2* allele. In the schematic graph, open and filled circles represent start codon and stop codon; light grey and dark grey boxes represent R2 and R3 domains. (c) The yeast two‐hybrid tests for the formation of the ternary complex, MBW by FaMYB10‐1 (red strawberry) and FaMYB10‐2 (white strawberry). Interactions of FaMYB10‐2, FaMYB10‐1, FaMYB10‐1‐N (1‐177 amino acids), FaMYB10‐1‐C (178‐233 amino acids), FaWD40 and PavBHLH with PavbHLH and PavWD40.

Since FaMYB1 and FaMYB10 play critical roles in controlling the coloration of strawberry fruits, the genes encoding these two transcriptional regulators were selected for further analysis in red and white strawberry varieties (Figure 4a). cDNAs of *FaMYB1* and *FaMYB10* were amplified separately from ripe fruits of red and white strawberry varieties using primers designed on the basis of predicted *FaMYB1* and *FaMYB10* sequences from *F. vesca* genomic sequences. Differences in cDNA sizes were examined by gel electrophoresis of the RT‐PCR products (Figure 4b), and the corresponding cDNA bands were excised from the gel and sequenced (Table 2). The *FaMYB1* gene produced only two transcript types, a full‐length transcript, *FaMYB1‐1* (564 bp), and a partial length transcript, *FaMYB1‐2* (561 bp) (Figure 4b). Comparing the *FaMYB1‐1* cDNA to the *FaMYB1‐2* cDNA, there was a deletion of three nucleotides (at position 441 from the initiating ATG) in *FaMYB1‐2*, leading to the predicted loss of one amino acid (Asp) in FaMYB1‐2, and a predicted protein of 186 amino acids (Figure 4b). Both *FaMYB1‐1* and *FaMYB1‐2* transcripts were found in WS and RS fruit with transcript ratios of 12:2 and 7:2, respectively (Table 2), suggesting that *FaMYB1‐1* and *FaMYB1‐2* alleles probably occur naturally in both octoploid strawberry varieties, in agreement with the similar expression patterns of *FaMYB1* in both varieties. Two transcripts for *FaMYB10* were also detected*,* presumably derived from different alleles, namely *FaMYB10‐1* (702 bp), encoding a full‐length protein, and *FaMYB10‐2* (710 bp), a transcript with an eight‐base insertion (ACTTATAC) relative to *FaMYB10‐1.* Interestingly, the *FaMYB10‐2* transcript was found only in 24 clones from WS, while RS (28 clones) contained only the *FaMYB10‐1* transcript (Table 2). Analysis of the coding sequence of *FaMYB10‐2* revealed that an 8‐bp (ACTTATAC) insertion occurred at position 491 in *FaMYB10‐2*, resulting in a premature stop codon and the predicted loss of 54 amino acids from the C‐terminus of the predicted FaMYB10 protein encoded by the *FaMYB10‐2* transcript (Figure 4b, 179 amino acid versus 233 amino acid). The predicted truncation of the protein may be the reason for the loss of fruit colour in WS.

**Table 2:** Comparison of *FaMYB1* and *FaMYB10* cDNA sequences in white and red strawberry varieties

Gene	Allele	White strawberry		Red strawberry	
		Size (bp)	Ratio	Size (bp)	Ratio
*FaMYB1*	*FaMYB1‐1*	564	12/14	564	7/9
	*FaMYB1‐2*	561	2/14	561	2/9
*FaMYB10*	*FaMYB10‐1*	–	–	702	28/28
	*FaMYB10‐2*	710	24/24	–	–

Ratio is the number of sequenced *FaMYB1* clones compared to the total number of *FaMYB1‐1* and *FaMYB1‐2* clones or the number of *FaMYB10* clones compared to *FaMYB10‐1* and *FaMYB10‐2* clones.

In the classic model of the regulation of anthocyanin and proanthocyanin biosynthesis, it has been proposed that there is a MBW complex consisting of MYB proteins, bHLH‐ and WD40‐repeat proteins. The MBW complex provides the core controlling hub for fruit coloration, through the transcriptional regulation of anthocyanin structural genes (D'Amelia *et al.*, 2014; Gonzalez *et al.*, 2008; Jin *et al.*, 2016; Schaart *et al.*, 2013). To test the formation of the MBW complex, FaMYB10‐1 and FaMYB10‐2 alleles from strawberry varieties were cloned into yeast expression vectors and examined in yeast two‐hybrid assays (Y2H assays) together with *FaWD40*, *PavbHLH*. Previous studies showed that PavMYB10.1a interacted with PavbHLH and PavWD40, but PavMYB10.1b did not interact with PavbHLH or PavWD40 by Y2H assays (Jin *et al.*, 2016). Hence, PavbHLH was chosen as a partner of *FaMYB10‐1* and *FaMYB10‐2* alleles. Y2H assays showed that FaMYB10‐1 was able to interact with two regulators, FaWD40 and PavbHLH, while FaMYB10‐2 could not (Figure 4c; Figure S5). To test which region of FaMYB10 protein was required for the formation of the MBW complex, truncated *FaMYB10‐1* constructs were designed, FaMYB10‐1‐N, which was shortened by loss of amino acids 178‐233 at the C‐terminus of the protein and FaMYB10‐1‐C, which was shortened by loss of amino acids 1–177 at the N‐terminus of the protein. Y2H assays showed that FaMYB10‐1‐N was able to interact with PavbHLH, while FaMYB10‐1‐C could not (Figure 4c; Figure S5), supporting previous reports that the interaction motif in R2R3MYB proteins lies within R3 of the N‐terminal DNA‐binding domain (Zimmermann *et al.*, 2004). β‐gal staining assays showed positive interactions in yeast containing pGADT7‐FaMYB10‐1 plus pGBKT7‐FaWD40, pGBKT7‐PavbHLH grown on ‐T/‐L/‐H/‐A screening medium, but no growth of yeast containing pGADT7‐FaMYB10‐2 plus pGBKT7‐FaWD40, pGBKT7‐PavBHLH or the empty pGBKT7 vector was observed. The results of β‐galactosidase activity tests were consistent with the idea that FaMYB10‐1 forms a canonical MBW complex with bHLH and WD40, but FaMYB10‐2 cannot form the MBW complex, indicating that the *FaMYB10‐2* allele from WS may be unable to induce anthocyanin biosynthesis, which may explain the fruit colour difference between RS and WS.

**Figure 5:**
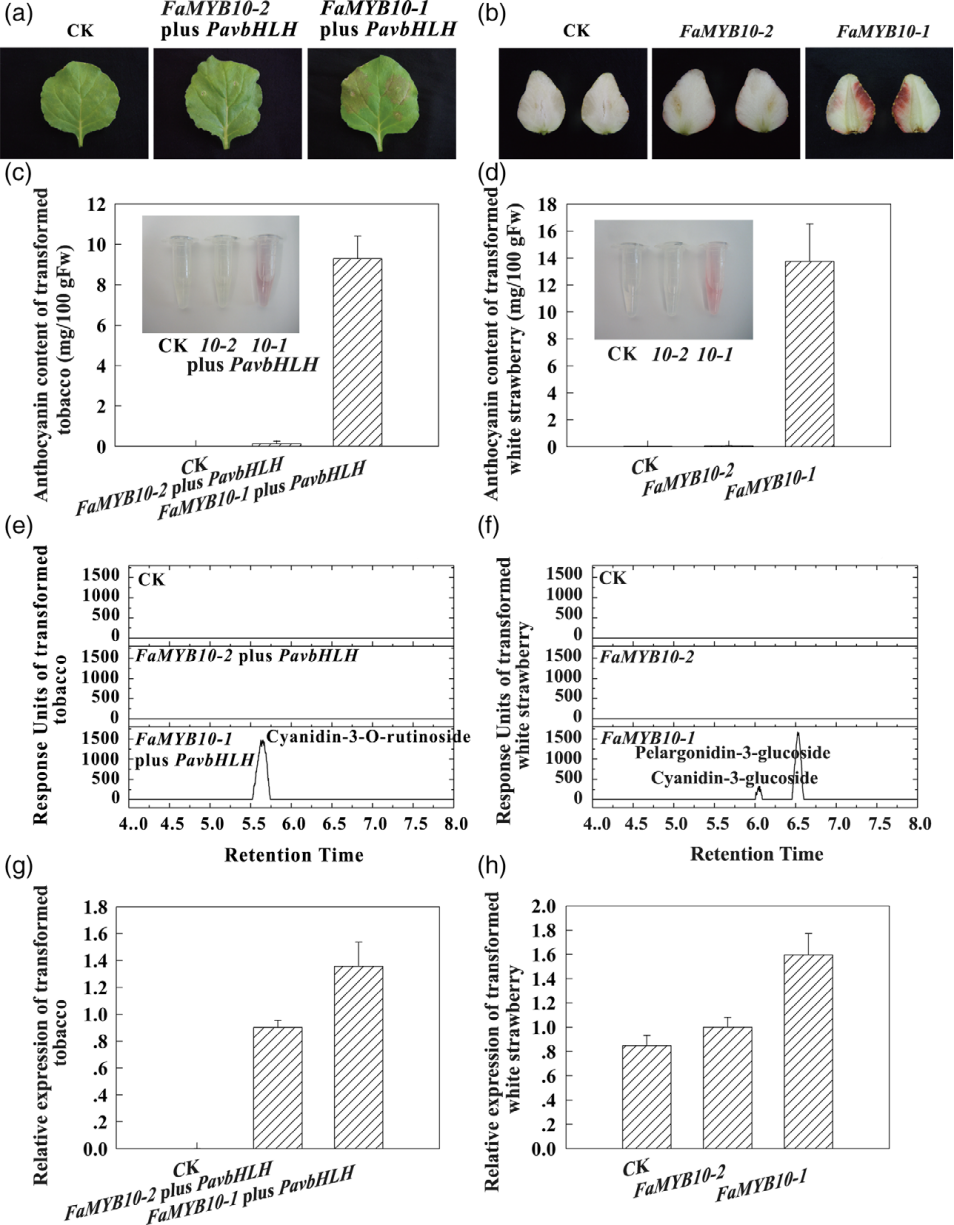
Transient over‐expression of *FaMYB10‐1* and *FaMYB10‐2*. Tobacco (*Nicotiana benthamiana*) leaves and flesh of white strawberry variety (‘Snow Princess’) were infiltrated with cDNA constructs corresponding to two different *FaMYB10* alleles and agroinfiltration solution (CK). (a) The phenotypes of leaves of tobacco after being infiltrated. (b) The phenotypes of fruits of WS after being infiltrated. (c) The anthocyanin contents of extracts of leaves of tobacco after being infiltrated. (d) The anthocyanin contents of extracts of fruits of WS after being infiltrated. (e) The chromatograms of anthocyanins in leaves of tobacco after being infiltrated. (f) The chromatograms of anthocyanins in fruits of WS after being infiltrated. (g) The expression analysis of *FaMYB10* in leaves of tobacco after being infiltrated. (h) The expression analysis of *FaMYB10* in fruits of WS after being infiltrated.

Transient transformation was used to confirm the function of the *FaMYB10* alleles. The HyperTrans expression system was used to deliver gene expression vectors transiently to the abaxial side of leaves of tobacco (*Nicotiana benthamiana*) and flesh of white strawberry at the W stage. Anthocyanins accumulated in leaves of tobacco infiltrated with the *FaMYB10‐1* expression vector plus *PavbHLH* expression vector and flesh of WS infiltrated with the *FaMYB10‐1* expression vector, while anthocyanin was absent with the *FaMYB10‐2* expression vector or agroinfiltration solution (CK) alone (Figure 5). After 5 days, anthocyanin accumulation was observed in the tobacco leaves infiltrated with the *FaMYB10‐1* expression vector plus *PavbHLH* expression vector and white fruit infiltrated with the *FaMYB10‐1* expression vector, which was not observed with the *FaMYB10‐2* expression vector or CK alone (Figure 5a and b). Anthocyanins accumulated to 9.30 mg/100g FW in the tobacco leaves and 13.74mg/100g FW in the white fruit infiltrated with the *FaMYB10‐1* expression vector, while anthocyanins were undetectable in leaves infiltrated with the *FaMYB10‐2* expression vector or CK (Figure 5c and d). The anthocyanin was identified as cyanidin‐3‐O‐rutinoside in the tobacco leaves and was mainly pelargonidin‐3‐glucoside in the white strawberry fruits infiltrated with the *FaMYB10‐1* expression vector (Figure 5e and f), confirming that the HyperTrans experiment had worked. *FaMYB10* showed striking differences in transcript levels in tobacco leaves and in the white strawberry fruits infiltrated with the *FaMYB10‐2* and *FaMYB10‐1* expression vectors, compared to CK (Figure 5g and h). These results confirmed that *FaMYB10* genes control anthocyanin and colour production in fruit of octoploid strawberry and that the loss of the functional FaMYB10 protein is critical to the loss of fruit colour in white octoploid strawberry.

### The genomic sequences of *FaMYB10* differ between WS and RS

**Figure 6:**
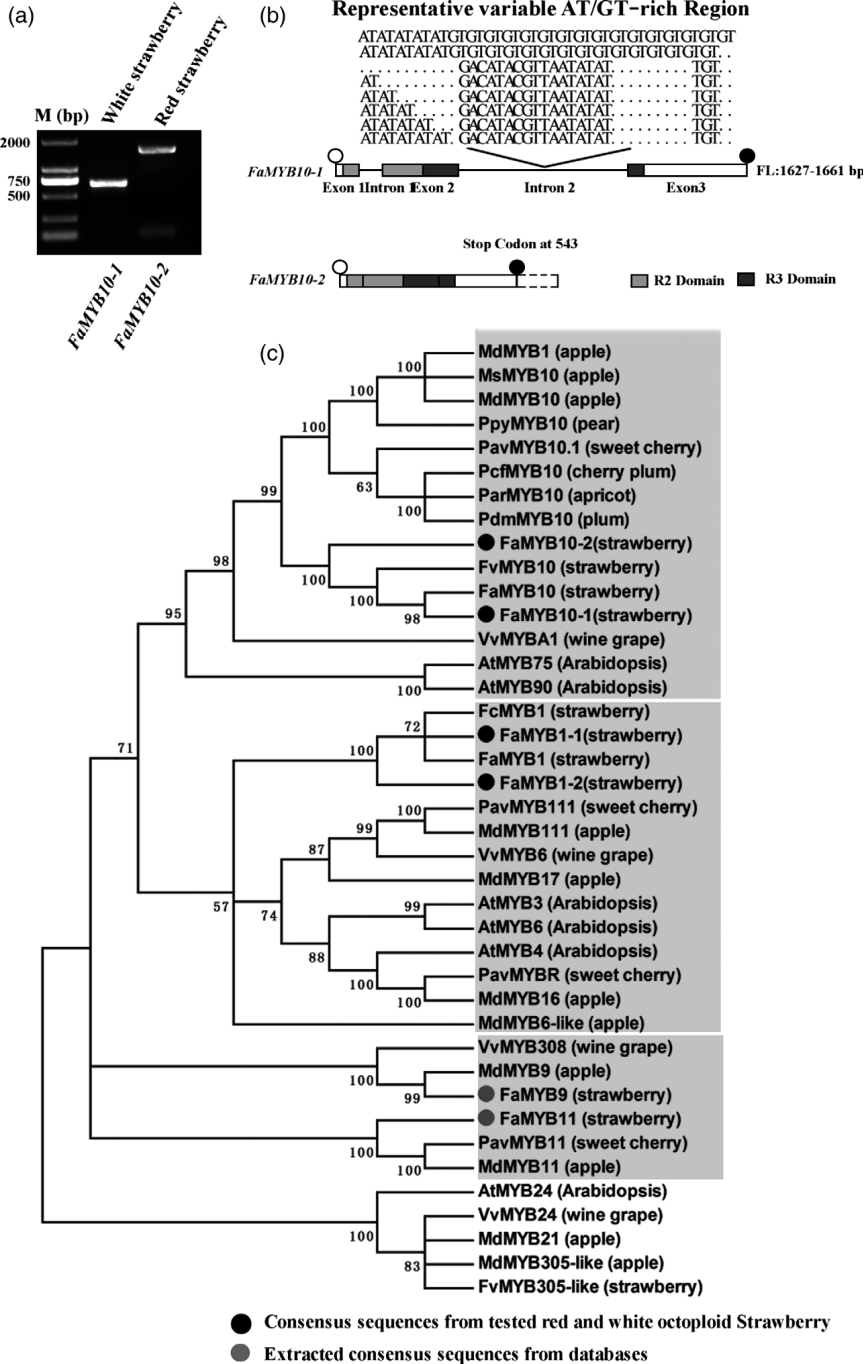
Genomic sequence analysis for *FaMYB10* alleles in red and white strawberry varieties and phylogenetic analysis of homologous transcriptional regulators. (a) The agarose gel of *FaMYB10* alleles. (b) Schematic graph for gene structure of *FaMYB10* alleles emphasizing the different regions of *F*a*MYB10* intron examined. (c) Phylogenetic analysis of the flavonoid‐related MYB transcriptional factors based on the amino acid sequences of the MYB proteins. Open and filled black circles represent start codon and stop codon, respectively, in the schematic graph. The grey and dark boxes represent the sequences from online databases and the sequenced *FaMYB* family gene alleles in the phylogenetic tree.

To investigate whether there were genomic differences between *FaMYB1* and *FaMYB10* alleles in RS and WS, genomic DNA was amplified by PCR, subcloned and sequenced. Eighty‐seven and 92 independent clones were selected from red and white octoploid strawberry varieties, respectively. Our results demonstrated that the sizes of *FaMYB1‐1* and *FaMYB1‐2* alleles varied from 1352 bp to 1534 bp (Figure S6; Table 3). The major difference in *FaMYB1* genomic sequences appeared in the second intron of *FaMYB1* alleles with a variable number of GA repeats (Figure S6), but the distribution of *FaMYB1* genomic sequence types was highly similar in the analysed clones between RS and WS (Table 3). However, this was not the case for the analysis of the *FaMYB10* genomic sequences. All the analysed genomic sequences of *FaMYB10‐1* contained two introns in positions consistent with the intron locations of other *R2R3MYB* genes (Jin *et al.*, 2016; Kobayashi *et al.*, 2004). The genes encoding FaMYB10‐1 varied in size from 1627 to 1661 bp due to differences in the length of intron 2 (Table 3). *FaMYB10*‐2 alleles were amplified solely from WS, and strikingly, all clones had no intron sequences (Figure 6a and b; Table 3).

**Table 3:** Comparison of *FaMYB1* and *FaMYB10* genomic sequences in white and red strawberry varieties

Gene	Type	White strawberry		Red strawberry	
		Size (bp)	Ratio	Size (bp)	Ratio
*FaMYB1*	*FaMYB1‐1*	1352	7/26	1352	10/61
		1475	1/26	1475	8/61
		1490	1/26	1490	2/61
		1501	5/26	1501	10/61
		1515	3/26	1515	7/61
		1534	7/26	1534	17/61
	*FaMYB1‐2*	1352	2/26	1352	2/61
		1515	–	1515	1/61
		1534	–	1534	4/61
*FaMYB10*	*FaMYB10‐1*	–	–	1627	3/72
		–	–	1629	3/72
		–	–	1631	7/72
		–	–	1633	16/72
		–	–	1635	27/72
		–	–	1637	4/72
		–	–	1659	2/72
		–	–	1661	10/72
	*FaMYB10‐2*	710	20/20	–	–

After the examination of coding regions in *FaMYB1* genomic sequences*,* if it contained the coding sequence of *FaMYB1‐1* or *FaMYB1‐2,* it was assigned as the genomic sequences for *FaMYB1‐1* or *FaMYB1‐2*; ratio shows the number of *FaMYB1‐1* or *FaMYB1‐2* clones to total sequenced clones; after examination of coding regions in *FaMYB10* genomic sequences*,* if it contains the coding region of *FaMYB10‐1* or *FaMYB10‐2*, it was assigned to the genomic sequences for *FaMYB10‐1* or *FaMYB10‐2*, respectively**;** ratio is the number of *FaMYB10‐1* or *FaMYB10‐2* clones to total sequenced clones.

### Phylogenetic analysis of homologous transcriptional regulators

To establish the molecular evolutionary relationship among the colour‐related *MYB* genes from different plant species, a phylogenetic tree was constructed by the neighbour‐joining method using 40 closely related R2R3‐type *MYB* genes including strawberry *FaMYB1* alleles and *FaMYB10* alleles, based on the full‐length deduced amino acid sequences (Figure 6c). The protein products of *FaMYB10‐1* and *FaMYB10‐2* clustered with all known *MYB10*‐type proteins that regulate anthocyanin biosynthesis, including *MdMYB1*, *AtMYB75* (production of anthocyanin pigment 1 protein, PAP1), *AtMYB90* and *VvMYBA1*, which grouped in a single clade. In previous studies, *MdMYB10*, *VvMYBA1* and *PavMYB10.1* have been demonstrated to control fruit skin colours by controlling anthocyanin biosynthesis (Espley *et al.*, 2007; Jin *et al.*, 2016; Kobayashi *et al.*, 2004), suggesting that *FaMYB10* has a very similar, if not identical, function in the regulation of fruit coloration through controlling expression of anthocyanin biosynthetic genes in octoploid strawberry (Figure 6c). Secondly, FaMYB1‐1 and FaMYB1‐2 clustered with other *MYB1*‐type proteins from different plant species AtMYB6‐like, MdMYB17, MdMYB111, PavMYB111 and VvMYB6 as a single independent clade; FaMYB9 and FaMYB11 cluster in the same clade together with MdMYB9, MdMYB11 and VvMYB308, suggesting that FaMYB9 and FaMYB11 are involved in the control of proanthocyanin biosynthesis similar to MdMYB9 and MdMYB11 in other species (An *et al.*, 2015; Schaart *et al.*, 2013).

## Discussion

### A regulatory model for the production of flavonoids in octoploid strawberry fruit

In recent years, there has been considerable progress in understanding the molecular mechanisms underpinning the coloration of fruit and flowers through multiple investigations of transcriptional regulation of key genes (Allan *et al.*, 2008; Martin *et al.*, 2010; Schwinn *et al.*, 2014). It has been found that R2R3MYB TFs play critical regulatory roles in determining the accumulation of anthocyanins in several horticultural crops (Jin *et al.*, 2016; Kobayashi *et al.*, 2004; Zhang *et al.*, 2014). In comparison with the known *FaMYB* genes extracted from the databases, the putative *FaMYB1‐1* allele and *FaMYB10‐1* allele from RS were included for the analysis of R2R3 domain similarity using conserved regions of homology. The coding sequences of *FaMYB1‐1*, *FaMYB9*, *FaMYB11* and *FaMYB10‐1* all encoded intact R2 and R3 domains (Table 4). Among these *FaMYB* genes, the shared similarity of the coding sequences in their R2 and R3 domains ranged from 58.3% to 82.6%, but there was very little similarity in their encoded C‐terminal regions ranging from 6.6% to 28.6% (Table 4). These levels of similarity suggest that *FaMYB1‐1*, *FaMYB9*, *FaMYB11* and *FaMYB10‐1* may interact only partially with the same binding partners in the MBW complex (Lin‐Wang *et al.*, 2014; Schaart *et al.*, 2013) and/or may recognize distinct DNA‐binding motifs in their target genes.

**Table 4:** Amino acid similarity analysis between the coding sequences of known *FaMYB* genes and the deduced coding sequence of *FaMYB10‐1* from octoploid red strawberry variety

Percentage Similarity (%)	N‐terminus	R2 domain	R3 domain	C‐terminus
FaMYB1‐1 vs FaMYB10‐1	14.3	58.3	82.6	6.6
FaMYB9 vs FaMYB10‐1	28.6	60.4	73.9	7.1
FaMYB11 vs FaMYB10‐1	28.6	60.4	76.1	9.4

To dissect further the network of transcriptional regulation in fruit coloration of strawberry varieties, principal component analysis (PCA) was used to decipher the interrelationships between the major *FaMYB* TFs and the structural genes of flavonoid biosynthesis in response to changes in endogenous ABA levels. PCA is often used to interpret the underlying relationships between multiple variables and reduces the dimensionality of a data set to a few composite variables that retain most of the information in the original variables (Jin *et al.*, 2012; O'Brien *et al.*, 2004; Wang *et al.*, 2012). In the current PCA, the factor loadings mirrored the interrelationships exhibited by anthocyanins, flavonols, proanthocyanidins, transcriptional levels of regulatory and structural genes, and endogenous ABA content in RS and WS at the key developmental stages for fruit coloration, the T and R stages (Figure S7). Two PCA components explained 80.32% of the variance in fruit coloration of red and white strawberry varieties. The first axis explained 55.29% of the variance and was positively correlated with pelargonidin‐3‐glucoside, the transcriptional changes in *FaMYB1*, *FaMYB10*, *FaCHS*, *FaCHI*, *FaF3H*, *FaANS* and *FaUFGT* and the changes in endogenous ABA levels, indicating that the variation in transcript levels of *FaMYB1* and *FaMYB10* influenced directly anthocyanin accumulation in response to the high concentrations of endogenous ABA, in accordance with the up‐regulation of expression of key transcriptional factors and structural genes controlling anthocyanin biosynthesis. This axis is referred to hereafter as the ‘anthocyanidin index’ (Figure S7). The second axis explained an additional 25.03% of the variance and was positively correlated with the concentrations of proanthocyanins and proanthocyanin precursors and the transcriptional variation of *FaMYB9*, *FaMYB11*, *FaLAR* and *FaFLS*, in accordance with key structural and transcription factor genes for the biosynthesis of proanthocyanins and proanthocyanin precursors, indicating that the accumulation of proanthocyanins was closely regulated by *FaMYB9* and *FaMYB11*. This axis is referred to hereafter as the ‘proanthocyanin index’ (Figure S7). Finally, the PCA showed that the accumulation of anthocyanins was, in general, related to the variation of endogenous ABA levels at different maturation stages in strawberry fruit.

Abscisic acid (ABA) is an important hormone associated with fruit maturation processes, such as sugar accumulation, softening, and coloration in sweet cherry (Ayub *et al.*, 2016; Jin *et al.*, 2016; Ren *et al.*, 2010; Shen *et al.*, 2014). In our study, at the early developmental stages, from the G1 to T stages, the level of endogenous ABA was maintained at relatively low levels, compared to R and OR stages in both RS and WS varieties, although the level of endogenous ABA was significantly higher in RS, in accordance with the increase in the levels of transcripts of the key structural genes, *FaLAR*, and *FaANR* for proanthocyanin biosynthesis and the higher concentrations of proanthocyanins observed in RS. In contrast, *FaMYB10* was expressed at a low level in both strawberry varieties at the early developmental stages, which may explain the low anthocyanin content at these stages. During fruit maturation of strawberry, the endogenous ABA appeared significantly higher from T to OR stages, in accordance with the increase in the transcripts of *FaMYB1* and *FaMYB10*. TFs *FaMYB1* and *FaMYB10* activated the transcripts of the key EBG genes, including *FaPAL*, *FaCHS*, *FaCHI* and *Fa4CL* to supply the precursors of flavonoid biosynthesis and the key LBG genes, including *FaANS*, *FaUFGT* and *FaDFR* for anthocyanin biosynthesis. Interestingly, *FaMYB1* had been shown to be negatively correlated with anthocyanin biosynthesis, but the results of our PCA and phylogenetic analysis suggested positive functions for *FaMYB1*, which may include a partial role in promoting the formation of anthocyanin in different tissues, as observed in red strawberry seeds in WS (Figure S1a). Furthermore, *FaMYB9/11* are not able to clearly cluster with the key structural genes and the key flavonoid components, suggesting that these *R2R3FaMYB* genes are probably suppressed in the later developmental stages of strawberry fruit (Schaart *et al.*, 2013).

**Figure 7:**
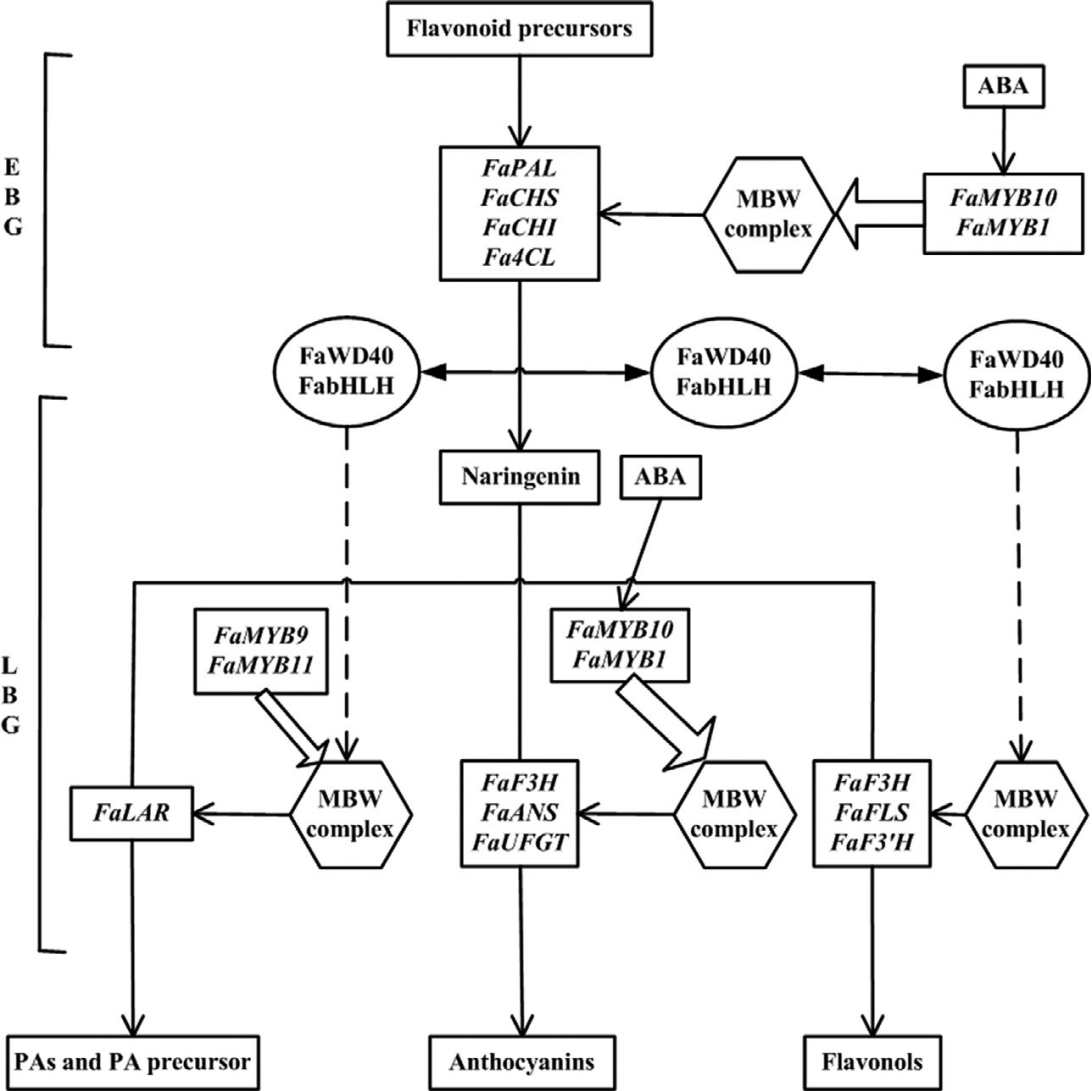
The proposed model for combinatorial effects of *FaMYB1*, *FaMYB10*, *FaMYB9* and *FaMYB11* on the regulation of the flavonoid biosynthetic pathways in response to endogenous ABA maturation signal. ABA is a signal molecule that promotes *FaMYB10* and *FaMYB1* expressions, and FaMYB10 or FaMYB1 interacts with bHLH and WD40 to form a putative canonical MBW activation complex, which activates transcription of structural genes and causes anthocyanin accumulation. Moreover, FaMYB9 or FaMYB11 interacts with bHLH and WD40 to form a MBW complex, which activates transcription of structural genes and causes accumulation of proanthocyanins.

A regulatory model for major flavonoid components was derived from the above experimental analyses. This model depicts the interrelationships of the key *FaMYB* genes in regulating the composition of flavonoid components through the differential transcription of key structural genes for anthocyanin, proanthocyanin and flavonol biosynthesis at the late stages of strawberry fruit development (Figure 7). ABA is a signal molecule that promotes *FaMYB10* and *FaMYB1* expressions, and FaMYB10 or FaMYB1 interacts with bHLH and WD40 to form a putative canonical MBW activation complex, which activates transcription of structural genes and causes anthocyanin accumulation. Moreover, FaMYB9 or FaMYB11 interacts with bHLH and WD40 to form a MBW complex, which activates transcription of structural genes and causes accumulation of proanthocyanins.

### The R2R3 MYBs control fruit colour, and *FaMYB10‐2* leads to coloration loss in the fruits of WS

In many fruits, R2R3 MYB TFs control the expression of structural genes in the anthocyanin biosynthetic pathway. *MYB* genes play an important role in anthocyanin accumulation in fruit. In sweet cherry, an R2R3MYB PavMYB10.1 controls fruit skin colour. Three functional alleles of the gene, *PavMYB10.1a*, *PavMYB10.1b* and *PavMYB10.1c*, lead to different fruit colours. *PavMYB10.1a* is dominant to *PavMYB10.1b*, and *PavMYB10.1b* is dominant to *PavMYB10.1c*. The allele *PavMYB10.1a* is present in sweet cherry with dark red fruit, but not in those with yellow fruit, while the allele *PavMYB10.1b* is present in sweet cherry with blush fruit, but not in those with yellow fruit. Only the *PavMYB10.1c* allele produced yellow fruit (Jin *et al.*, 2016). In Citrus, an R2R3 MYB *Ruby*, a transcriptional activator of anthocyanin production, controls fruit colour. Sicilian blood oranges (*Citrus sinensis*) arose by insertion of a Copia‐like retrotransposon adjacent to the *R2R3MYB* gene encoding Ruby. The retrotransposon induces the expression of the *Ruby* gene in fruit. In different accessions of *Citrus* species and in domesticated cultivars, the differences in transcript levels of the *Ruby* caused by point mutations, deletions and insertions of transposable elements lead to natural variation in leaf and flower colours (Butelli *et al.*, 2012, 2017). In apple (*Malus domestica*), R2R3 MYB TFs responsible for anthocyanin accumulation in fruit are encoded by *MdMYB1*, *MdMYB10* and *MdMYBA* transcripts, which are likely to be allelic. The promoter of *MdMYB10* has an allelic rearrangement that regulates *MdMYB10* transcript levels and the subsequent ectopic accumulation of anthocyanins (Allan *et al.*, 2008; Ban *et al.*, 2007; Espley *et al.*, 2007; Lin‐Wang *et al.*, 2010; Takos *et al.*, 2006; Telias *et al.*, 2011).

In strawberry, *F. vesca* is a diploid species of wild strawberry. A candidate SNP (single nucleotide polymorphism) in *FvMYB10* was functionally confirmed to be responsible for yellow‐coloured fruits. The specific SNP (G to C) exhibits a Trp to Ser within the R2 DNA‐binding domain of FaMYB10. All red accessions have G, while all yellow accessions have C (Hawkins et al., 2016). However, we found the conserved G at the R2 DNA‐binding domain in FaMYB10 in octoploid RS and WS. Through the analysis of the sequences of different *FaMYB10* alleles, RS carries allele(s) encoding a full‐length transcript, *FaMYB10‐1*, whereas WS carries only allele(s) with the insertion, which encode a predicted protein with an early termination codon. This truncated FaMYB10 protein was shown to be non‐functional in that it was defective in forming an MBW complex and in activating anthocyanin biosynthesis in *N. benthamiana*. The insertion, ACTTATAC, in the region of the *FaMYB10* gene encoding the C‐terminus of the FaMYB10 protein may explain the loss of anthocyanin biosynthesis in WS due to the loss of transcriptional activation by the MBW complex. The *FaMYB10‐2* allele lacks introns, suggesting that it was generated by reverse transcription, in a process that might also have involved transposition. Based on *N. benthamiana* transient over‐expression of different *FaMYB10* alleles, the full‐length *FaMYB10‐1* cDNA could induce the accumulation of anthocyanins to visible levels but *FaMYB10‐2* could not. Similarly, in transient over‐expression of different *FaMYB10* alleles in WS, the full‐length *FaMYB10‐1* cDNA developed red pigmentation and complemented the WS phenotype, whereas *FaMYB10‐2* did not. These results show that the mutation in *FaMYB10‐2* leads to the loss of coloration in the fruits of WS.

## Experimental procedures

### Plant materials and growth conditions

Red octoploid strawberry variety (*Fragaria* × *ananassa* ‘Sweet Charlie’, red strawberry; Chandler *et al.*, 1997) and white octoploid strawberry variety (*F.* × *ananassa* ‘Snow Princess’, white strawberry; Zhang *et al.*, 2016) were grown in a greenhouse at the Beijing Academy of Forestry and Pomology Sciences, China. Plant tissues were collected for DNA extraction for gene cloning, for RNA for quantitative PCR and for flavonoid analyses. Based on the seed and fruit colour of ‘Snow Princess’, six visual developmental stages were defined at 13, 17, 24, 30, 46 and 50 day after anthesis. Based on the seed and fruit colour of ‘Sweet Charlie’, six visual developmental stages were defined as: small green fruit stage (G1), big green fruit stage (G2), white fruit stage (W), turning fruit stage (T), ripe fruit stage (R) and over‐ripe fruit stage (OR) at 7, 12, 17, 19, 26 and 30 day after anthesis, as shown in Figure S1 (Moyano *et al.*, 1998; Schaart *et al.*, 2013). For obtaining the experiment material from strawberry fruits, all seeds were removed from the fruit before extraction; fully expanded leaves and fruits of different developmental stages were harvested, immediately frozen in liquid nitrogen and stored at −80 °C until the analysis was performed.

### Measurement of total anthocyanin content

Fruits at different developmental stages of both white and red strawberry varieties were harvested, ground and extracted with the extraction solution (1% HCl in methanol) at room temperature and then in the dark for 4 h. The fruit extract was centrifuged at 8500 ***g*** for 20 min; the supernatant was collected and transferred to a clean tube and filtered through a 0.2‐μm filter. The total content of anthocyanins was estimated by a pH differential method (Benvenuti *et al.*, 2004; Cheng and Breen, 1991). The total anthocyanin content of individual experimental samples was calculated and estimated as cyanidin‐3‐glucoside equivalents mg per 100 g fresh weight (FW). All analyses were performed with three biological replicates.

### Tentative identification and quantification of flavonoid components

The analytical method was modified for the identification and quantification of flavonoid composition, anthocyanins, flavonols and proanthocyanins from strawberry fruit extracts in the fruits of red and white strawberry varieties at T and R stages (Veberic *et al.*, 2015). 10 mg of lyophilized fruit powder was extracted in 1mL of 0.1% acetic acid/methanol solution at 4 °C overnight for different materials at the T and R stage and centrifuged for 10 min at 14 500 ***g***, and then, the supernatant was collected for drying with a vacuum centrifuge concentrator (CV100‐DNA, Baijiu, Beijing, China). The dried experimental materials were stored at −20 °C before analysis. The dried extracts were re‐dissolved in methyl alcohol before analyses and were examined with a ultra‐high‐performance liquid chromatography–mass spectrometry (UPLC‐MS/MS, Waters, Milford, MA) coupled to a triple‐Quadrupole Mass Spectrometry (XEVO®‐TQ) with electrospray ionization (ESI). The separation was carried out with a ZORBAX Eclipse plus C18 (150 mm × 3.0 mm) with particle size of 1.8 µm (Agilent Technologies, Santa Clara, California) at 40°C. The gradient was using 0.1% formic acid (A) and acetonitrile (B) as the mobile phases, 0–1 min (5% B), 1–8 min (5%–30% B), 8‐12 min (40%–95% B), 16‐17 min (95%–100% B), 17–21 min (100% B) and 21–25 min (5% B). The operating conditions were as follows: a flow rate of 0.4 mL/min and positive ion ESI modes, the capillary voltages of 3.0 kV and 16 L/h of nebulization nitrogen flow. The chromatographs were plotted and analysed using OriginPro 2015 (OriginLab Corporation, Northampton, MA).

Compounds were identified by comparison with the retention times of standards, the characteristics of UV‐Vis spectra of peaks and the mass spectrometric information with MassLynx V4.1 software (Waters, Milford, MA). The relative quantification of anthocyanins, flavonoids and proanthocyanins contents was calculated from peak areas of samples based on the intensity of the corresponding standard compounds, including quercetin‐3‐glucoside, kaempferol‐3‐rutinoside, cyanidin‐3,5 ‐diglucoside, pelargonidin‐3‐glucoside, proanthocyanidin B1 and proanthocyanidins, catechin and epicatechin. For compounds lacking corresponding standards, the quantification was carried out using similar compounds. All analysis was performed with three biological replicates.

### RNA‐seq analysis

Total RNA was isolated from the fruits of red and white strawberry varieties at T and R stages using an RNA isolation kit (Waryong, Beijing, China). Differential expression of flavonoid biosynthesis genes was analysed using the RPKM method, and the scaled expression levels were displayed as heatmaps (Mortazavi *et al.*, 2008). After the RNA extract had been treated with DNase I, first‐strand cDNA was synthesized with a RevertAid First‐Strand cDNA synthesis kit (Thermo Scientific Inc., MA). A total of 3 μg RNA per sample was used as input material for the RNA sample preparation at T and R stages from the white and red strawberry varieties. Sequencing libraries were generated using NEBNext® Ultra™ RNA LibraryPrep Kit for Illumina® (New England Biolabs, MA) according to the manufacturer's manual. The libraries were sequenced using Illumina HiSeq^™^ 2000 (Illumina incorporation, San Diego, California). The raw reads were first filtered by removing the adapter sequences and low‐quality sequences and aligned against *F. vesca* genome sequences. Functional UniGenes were predicted using BLAST program with *E*‐value threshold of 10^−5^ to protein databases, including the NCBI non‐redundant (nr) database, the Swiss‐Prot protein database, the Kyoto Encyclopedia of Genes and Genomes database (KEGG) and the Clusters of Orthologous Groups of proteins database (COG/KOG). Differential gene expression analyses were performed using RPKM method, and the scaled expression levels of flavonoid biosynthesis‐related transcription factors and structural genes were displayed as the heatmaps for the samples from the developmental stages, T and R, in both white and red strawberry varieties (Mortazavi *et al.*, 2008). The resulting *P* values were adjusted using the Benjamini and Hochberg's approach for controlling the false discovery rate. Genes with an adjusted *P*‐value < 0.05 found by DESeq were assigned as differentially expressed. All RNA‐seq analyses were performed using three biological replicates.

### Expression analysis of structural genes and transcription factors related to flavonoid biosynthesis

Expression analysis of flavonoid biosynthesis‐related genes was undertaken as described previously (Jin *et al.*, 2016; Livak and Schmittgen, 2001). Total RNA and the first‐strand cDNA were isolated and amplified from different developmental stages of strawberry fruits from G1 to OR. To further investigate various structural genes and transcription factors in the pathway of flavonoid biosynthesis during fruit development, specific primers were designed with Primer 5.0 program (Premier Biosoft International, Palo Alto, CA), as shown in Supplemental Table S6, and acquired from Sangon Inc. (Sangon, Shanghai, China). qRT‐PCR was conducted with on a Bio‐Rad CFX96 Real‐Time PCR Detection System (Bio‐Rad, CA) using 10 μL reaction mixture, which included 5 μL 2 × SYBR Premix (Bio‐Rad, CA), 1 μL forward primer (10 μm), 1 μL reverse primer (10 μm), 1 μL cDNA template (20 ng) and 2 μL ddH_2_O. PCR conditions were as follows: 1 cycle at 95 °C for 30 s, 40 cycles at 95 °C for 5 s and 1 cycle at 60 °C for 30 s. Finally, the transcription levels of different genes were analysed using the 2^−ΔΔCT^ method (Livak and Schmittgen, 2001). All analyses were performed with three biological replicates.

### cDNA and genomic sequence examination of *FaMYB1* and *FaMYB10* alleles

cDNA and genomic sequence examination of *FaMYB1* and *FaMYB10* alleles was undertaken as described previously (Jin *et al.*, 2016). Total cDNA was acquired, and total genomic DNA was extracted from 250 mg of fresh leaves with an EZ Spin Column Genomic DNA Isolation Kit (Waryong, Beijing, China). The PCR primers for *FaMYB1* and *FaMYB10* cDNAs and genomic DNAs were designed with the Primer 5.0 program (Premier Biosoft International, Palo Alto, CA), as shown in Table S6, and acquired from Sangon Inc. (Sangon, Shanghai, China).

For cDNA PCR conditions, 10 μL reaction mixture included 5 μL 2 × SYBR Premix, 1 μL forward primer (10 μm), 1 μL reverse primer (10 μm), 1 μL cDNA template (20 ng) and 2 μL ddH_2_O; the PCR conditions were as follows: 1 cycle at 95 °C for 30 s, 40 cycles at 95 °C for 5 s and 1 cycle at 60 °C for 30 s.

For genomic DNA PCR conditions, 20 μL PCR mixture included 2 μL 2 × buffer, 1 μL dNTPs (10 mmol), 1 μL primer (10 pmol), 2.5 U Taq polymerase (Sangon, Shanghai, China) and 3 μL genomic DNA (100 ng); the PCR condition was as follows: 1 cycle at 94 °C for 4 min, 35 cycles at 94 °C for 30 s, 55 °C for 30 s and 72 °C for 2 min, followed by a final cycle at 72 °C for 10 min. The PCR products were examined on 1% (w/v) agarose gels, stained with ethidium bromide (EtBr) and visualized with a gel imaging system (Gel Doc XR, Bio‐Rad, CA).

The amplified *FaMYB1* and *FaMYB10* cDNA and genomic DNA were cut from gels according to their predicted cDNA and genomic DNA sizes, and purified using an EZ‐10 Spin Column DNA gel extraction kit (Bio Basic Inc., ON, Canada), and then were cloned into pGEM‐T Easy (Promega, Madison, WI) for amplifying in *Escherichia coli* strain DH5α. Finally, *FaMYB1* and *FaMYB10* genes were sequenced.

### Yeast two‐hybrid (Y2H) assays

Y2H assays were described previously (Jin *et al.*, 2016). Yeast AH109 cells were co‐transformed with a specific bait and prey constructs according to the manufacturer's instructions (Clontech, CA). For Y2H assays of interactions of FaMYB10 (FaMYB10‐1 and FaMYB10‐2) with two regulators (FaWD40 and PavbHLH), *FaMYB10‐1*, *FaMYB10‐2*, *FaMYB10‐1‐N* (1–531 bp), *FaMYB10‐1‐C* (532–702 bp), FaWD40 and PavBHLH were amplified and cloned into pGADT7 vector (Clontech, CA); two regulators (FaWD40 and PavBHLH) of the flavonoid biosynthetic pathway were cloned into the pGBKT7 vector (Clontech, CA). All primers are listed in Table S6 and were acquired from Sangon Inc. (Sangon, Shanghai, China). All the constructs were confirmed by sequencing and then transformed into the yeast strain AH109 using the lithium acetate method (Gietz *et al.*, 1995). The subsequent clones were plated onto selective medium lacking Leu and Trp (Leu, leucine; Trp, tryptophan), and finally, putative transformants were transferred onto selective medium lacking Ade, His, Leu and Trp (Ade, adenine; His, histidine). After the selected colonies had been tested, β‐galactosidase assays were undertaken following a yeast protocol (PT3024‐1; Clontech, CA). The selected colonies were transferred to plates containing 40 μg/mL X‐α‐gal (Clontech, CA), and the vector P53 plus SV40 was used to exclude false‐positive activation.

### Transient expression in *Nicotiana benthamiana* leaves and ‘Snow Princess’ fruits

Transient expression of *FaMYB10‐1* and *FaMYB10‐2* cDNA constructs was carried out using the HyperTrans system (Butelli *et al.*, 2017; Sainsbury *et al.*, 2009). *FaMYB10* cDNAs were isolated from the flesh of red octoploid strawberry variety (‘Sweet Charlie’) and the white octoploid strawberry variety (‘Snow Princess’) at the R stage using an RNA isolation kit (Waryong, Beijing, China). PCR amplified (using primers listed in Table S6) was cloned into pEAQ‐HT vectors. The plasmids obtained were introduced into *Agrobacterium tumefaciens* GV3101. Each fresh colony was inoculated into 10 mL LB with Kan (50 μg/mL) and Rif (50 μg/mL) and grown at 28 °C overnight and then was spun down for 15 min at 4200 ***g***. The supernatant was removed completely, and cells were resuspended in 15 mL agroinfiltration solution (10 mm MgCl_2_, 10 mm MES pH 5.6). Cultures were spun down for 15 min at 4200 ***g***. The supernatant was removed completely, and cells were resuspended in 10 mL agroinfiltration solution (10 mm MgCl_2_, 10 mm MES pH 5.6) + 200 μm acetosyringone. Cultures were incubated for 2 h and then diluted to an OD_600_ of around 0.2. Then, the strains, including agroinfiltration solution (CK), *FaMYB10‐1* expression vector plus *PavbHLH* expression vector, *FaMYB10‐2* expression vector plus *PavbHLH* expression vector, were infiltrated into the abaxial side of leaves of five‐week‐old plants. Leaves were harvested 7 days after infiltration. The strains, including agroinfiltration solution (CK), *FaMYB10‐1* expression vector, *FaMYB10‐2* expression vector, were infiltrated into the flesh of white octoploid strawberry variety at the W stage. Fruits were harvested 14 days after infiltration. Harvested leaves and fruits were subsequently used for photographs, measurement of total anthocyanin content, HPLC analysis of anthocyanins and expression analysis of the transcription factor *FaMYB10*.

### Phylogenetic analysis of different *MYB* genes from different plant species

To perform the phylogenetic analysis of flavonoid biosynthesis‐related *MYB* genes, the *MYB* gene sequences in octoploid strawberry were first acquired from NCBI database for *FaMYB9*, *FaMYB11*, *FaMYB1* and *FaMYB10* and our consensus sequences of *FaMYB1* and *FaMYB10* allele sequences were acquired from the alignment of the sequenced clones from red and white strawberry varieties using BioEdit (http://www.mbio.ncsu.edu/BioEdit/bioedit.html) and ClustalW (Chenna *et al.*, 2003). Furthermore, to process the previously identified and putative *MYB* classes of genes in strawberry‐related species, the gene sequences of the identified *MYB* genes were extracted from NCBI databases using the Blastx program, based on the highest identity of protein sequences to *FaMYB9*, *FaMYB11*, *FaMYB1* and *FaMYB10*, respectively. These species included several fruit plant species from the Rosaceae family, such as apple, sweet cherry, wine grape, pears, and *Arabidopsis thaliana*. There were 40 MYB genes chosen for the phylogenetic analysis in total (Table S7).

To identify the conserved DNA‐binding domains, R2 and R3, and to separate the N and C termini of key strawberry MYB genes, *FaMYB9*, *FaMYB11*, *FaMYB1* and *FaMYB10* genes were predicted and analysed using the Pfam website (http://pfam.xfam.org). The whole amino acid sequences of 40 MYB genes were aligned with ClustalW using MEGA 7.0 software package. Based on the alignment of deduced amino acid sequences, a phylogenetic tree was constructed with 1000 bootstrap replicates by the neighbour‐joining method (NJ) using the MEGA 7.0 software package.

### Principal component analysis (PCA) for flavonoid biosynthesis‐related factors

On PCA of flavonoid biosynthesis‐related factors, the analysed data included the scaled measurement of major flavonoids, the scaled transcriptional levels of flavonoid biosynthesis‐related genes and the scaled measurement of endogenous ABA within at least three independent repeats. The data were pre‐treated using a range scaling method (van den Berg *et al.*, 2006). This PCA method was employed to evaluate how explanatory variables and the levels of anthocyanins, flavonols and proanthocyanins were correlated with different structural flavonoid biosynthetic and regulatory genes in response to the changes in endogenous ABA levels at two critical fruit developmental stages, T stage and R stages. Firstly, the PCA factor scores were extracted from the analysed data representing all variables after reducing sixteen variables to two factors. Secondly, the corresponding loading plots were generated with the factor scores from the PCA and displayed as the PCA score plot using SPSS 16.0 (SPSS Inc., Chicago, USA).

## Conflict of interest

The authors declare no conflict of interest.

## Authors' contributions

W.J. and C.M. designed research; H.W., Y.Y., E.B., M.L., Z.X. and J.D. performed research; H.W., H.Z., J.L., A.W., Y.Z., J.L. and G.W. analysed data; W. J., C.M., H.W. and H.Z., wrote the paper.

## Supporting information


**Figure S1** Fruit developmental stages of white and red octoploid strawberry varieties.
**Figure S2** Chromatographs of anthocyanins, flavonols, and proanthocyanins.
**Figure S3** Transcript levels of structural genes and regulatory genes not included in Figure 3 in the white and red strawberry varieties.
**Figure S4** Transcript levels of regulatory genes in the white and red strawberry varieties.
**Figure S5** The constructs and results from yeast two‐hybrid assay of FaMYB10‐1 or FaMYB10‐2 with two regulators (FaWD40 and PavbHLH) for the formation of MBW ternary complex.
**Figure S6** Genomic sequence analysis for FaMYB1 alleles in red and white strawberry varieties.
**Figure S7** PCA of key flavonoid components against flavonoid biosynthetic structural genes and transcription‐related genes.
**Table S1** Analysis of flavonoid contents.
**Table S2** The expression of structural genes, FaANS, FaUFGT, and FaDFR in flavonoid biosynthesis in red and white strawberry varieties.
**Table S3** The expression of structural genes, FaLAR, FaANR, and FaFLS in flavonoid biosynthesis in red and white strawberry varieties.
**Table S4** The expression of regulatory genes, FaMYB1, FaMYB10, and FaWD40 of flavonoid biosynthesis in red and white strawberry varieties.
**Table S5** The expression of regulatory genes, FaMYB9, and FaMYB11 of flavonoid biosynthesis in red and white strawberry varieties.
**Table S6** The sequences of the oligonucleotide primers used in this work.
**Table S7** Information about the MYB protein sequences used for the construction of the neighbour joining tree.

## Data Availability

The *FaMYB1*, *FaMYB10* sequence data for cDNA are available in the GenBank database under the accession numbers MG456857, MG456858, MG456859 and MG456860. RNA‐seq data are available in the CNGB Nucleotide Sequence Archive (https://db.cngb.org/cnsa/) of China National GenBank (CNGB) database with accession number CNP0000406.
