# Supplementary material for: The control of red colour by a family of MYB transcription factors in octoploid strawberry (Fragaria × ananassa) fruits
Source: Plant Biotechnol J. 2019 Dec 12;18(5):1169–84. doi: 10.1111/pbi.13282 (PMC7152614; doi:10.1111/pbi.13282)
Supplement: Supplementary file 1 — Figure S1 Fruit developmental stages of white and red octoploid strawberry varieties. Figure S2 Chromatographs of anthocyanins, flavonols, and proanthocyanins. Figure S3 Transcript levels of structural genes and regulatory genes not included in Figure 3 in the white and red strawberry varieties. Figure S4 Transcript levels of regulatory genes in the white and red strawberry varieties. Figure S5 The constructs and results from yeast two‐hybrid assay of FaMYB10‐1 or FaMYB10‐2 with two regulators (FaWD40 and PavbHLH) for the formation of MBW ternary complex. Figure S6 Genomic sequence analysis for FaMYB1 alleles in red and white strawberry varieties. Figure S7 PCA of key flavonoid components against flavonoid biosynthetic structural genes and transcription‐related genes. Table S1 Analysis of flavonoid contents. Table S2 The expression of structural genes, FaANS, FaUFGT, and FaDFR in flavonoid biosynthesis in red and white strawberry varieties. Table S3 The expression of structural genes, FaLAR, FaANR, and FaFLS in flavonoid biosynthesis in red and white strawberry varieties. Table S4 The expression of regulatory genes, FaMYB1, FaMYB10, and FaWD40 of flavonoid biosynthesis in red and white strawberry varieties. Table S5 The expression of regulatory genes, FaMYB9, and FaMYB11 of flavonoid biosynthesis in red and white strawberry varieties. Table S6 The sequences of the oligonucleotide primers used in this work. Table S7 Information about the MYB protein sequences used for the construction of the neighbour joining tree. [file PBI-18-1169-s001.pdf]

Supporting information for

The control of red color by a family of MYB transcription factors in octoploid strawberry (*Fragaria* × *ananassa*) fruits

Wang et al.

Figures: S1-S7

Tables: S1-S7

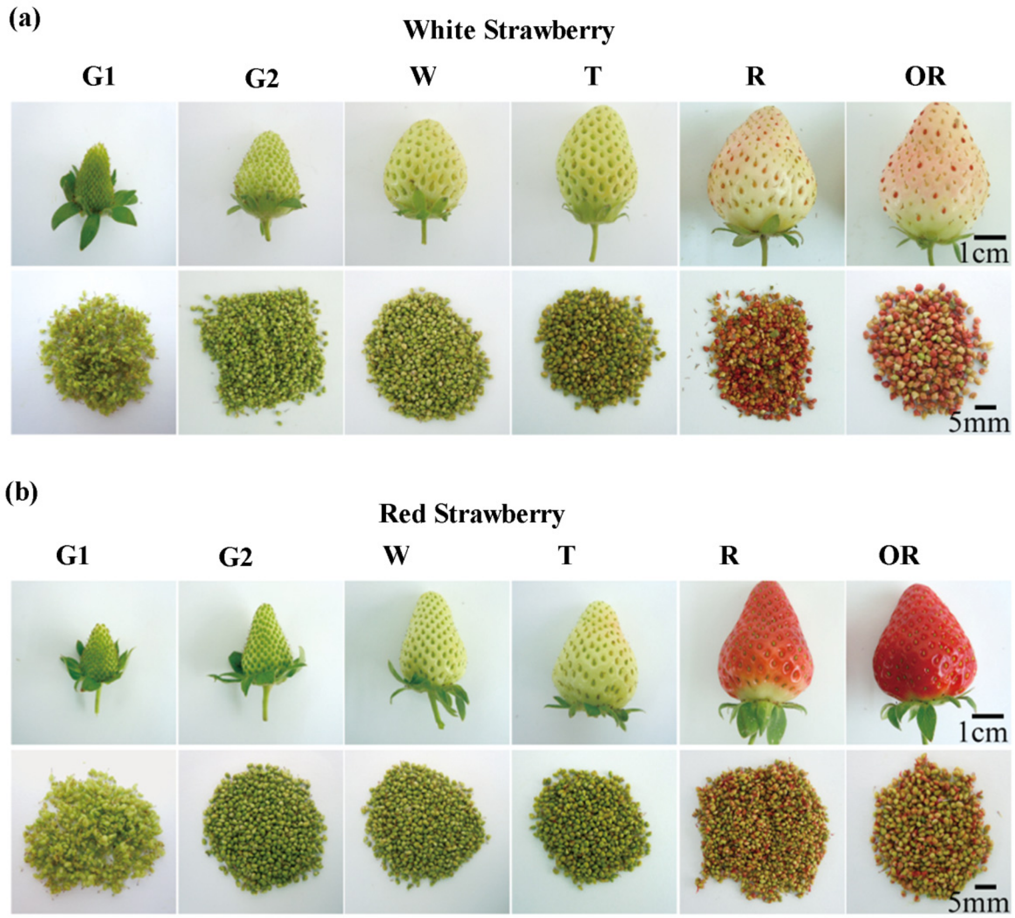

Figure S1 Friut developmental stages of white and red octoploid strawberry

(a) Based on the seed (lower row) and fruit colors (upper row) of the white octoploid strawberry, six visual developmental stages were defined as: small green fruit stage (G1), large green fruit stage (G2), white fruit stage (W), turning fruit stage (T), ripe fruit stage (R), and over-ripe fruit stage (OR) at 13, 17, 24, 30, 46, and 50 d after anthesis. (b) Based on the seed (lower row) and fruit colors (upper row) of red octoploid strawberry, six visual developmental stages were defined at 7, 12, 17, 19, 26, and 30 d after anthesis. Scale bars in (a, b), Fruit, 1 cm, Seed, 5mm.

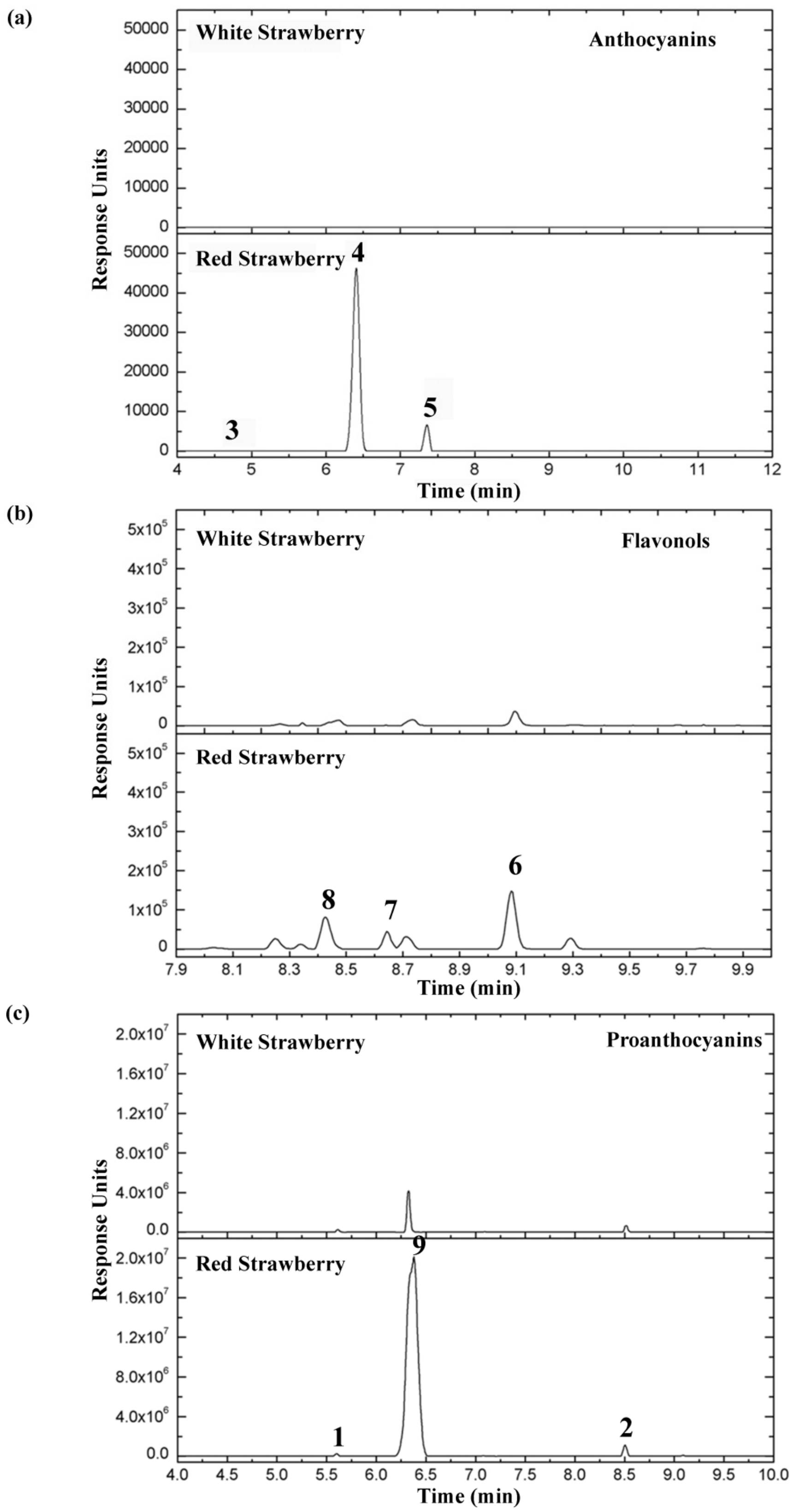

Figure S2 Chromatographs of anthocyanins, flavonols, and proanthocyanins. Chromatographic peaks were identified as shown in Table 1.

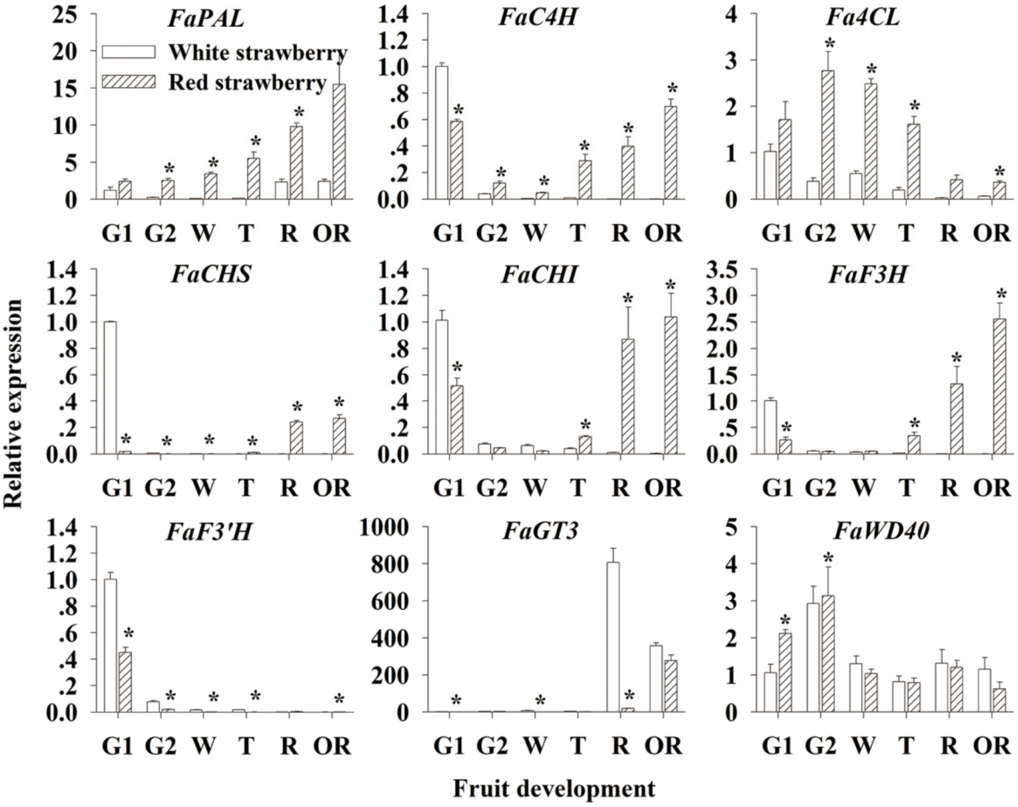

Figure S3 Transcript levels of structural genes and regulatory genes not included in Figure 3 in the white and red strawberry varieties. Asterisks (*) represent that the values of the corresponding transcript levels (n=3, ±SE) are significantly different at $p < 0.05$ as determined using independent t-test.

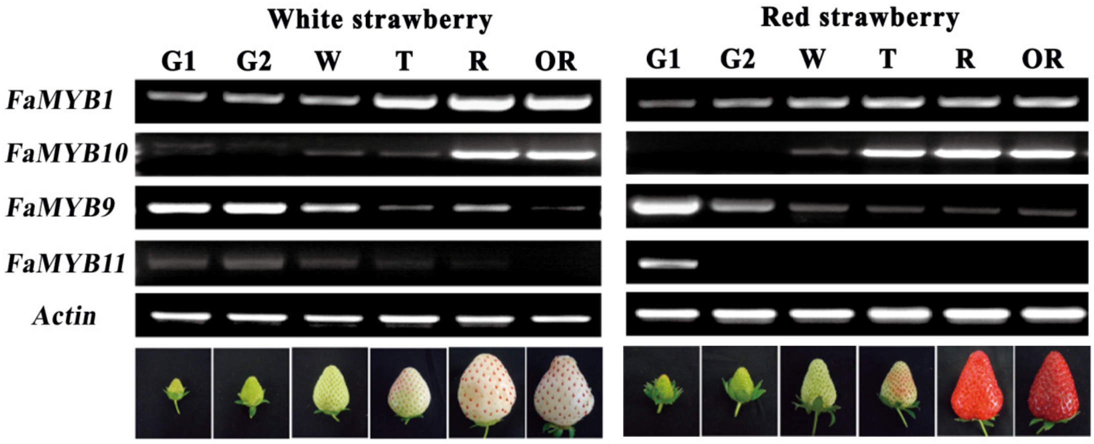

Figure S4 Transcript levels of regulatory genes in the white and red strawberry varieties. RT-PCR products were examined for *FaMYB1*, *FaMYB10*, *FaMYB9*, and *FaMYB11* transcripts on 1% (w/v) agarose gels, stained with EtBr. *Actin* was used as loading control. At the top, the corresponding developmental stages are shown as G1, G2, W, T, R, and OR with the fruit images of corresponding stages at the left, white strawberry variety and at the right, red strawberry variety.

(a)

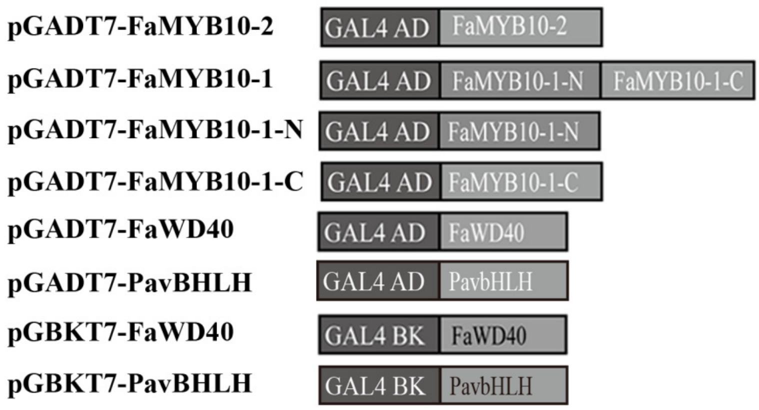

(b)

BK+ / AD+	FaWD40	PavbHLH	pGBKT7
FaMYB10-2			
FaMYB10-1	+	+	
FaMYB10-1-N		+	
FaMYB10-1-C			
FaWD40	+	+	
PavbHLH	+	+	
pGADT7			

Figure S5 The constructs and results from yeast two-hybrid assay of FaMYB10-1 or FaMYB10-2 with two regulators (FaWD40 and PavbHLH) for the formation of MBW ternary complex. (a) Schematics of FaMYB10-1, FaMYB10-2, FaWD40, and PavbHLH constructs used in yeast two-hybrid assays. (b) The results of interaction of FaMYB10-2, the different regions of FaMYB10-1 protein (the full-length coding region, the R2R3 MYB domain-containing N-terminal region, the C-terminal region) with FaWD40, PavbHLH.

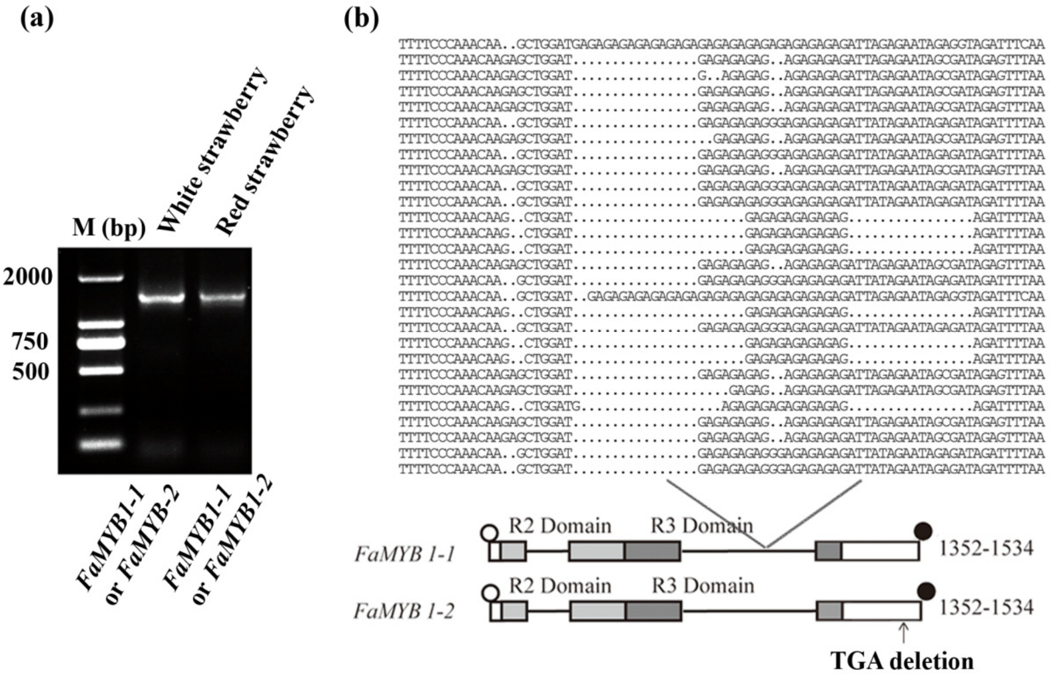

Figure S6 Genomic sequence analysis for *FaMYB1* alleles in red and white strawberry varieties. (a) The agarose gel of *FaMYB1* alleles. (b) Schematic graph for gene structure of *FaMYB1* alleles. Open and filled black circles represent start codon and stop codon; light gray and dark gray boxes represented R2 and R3 domains in the schemetic graph.

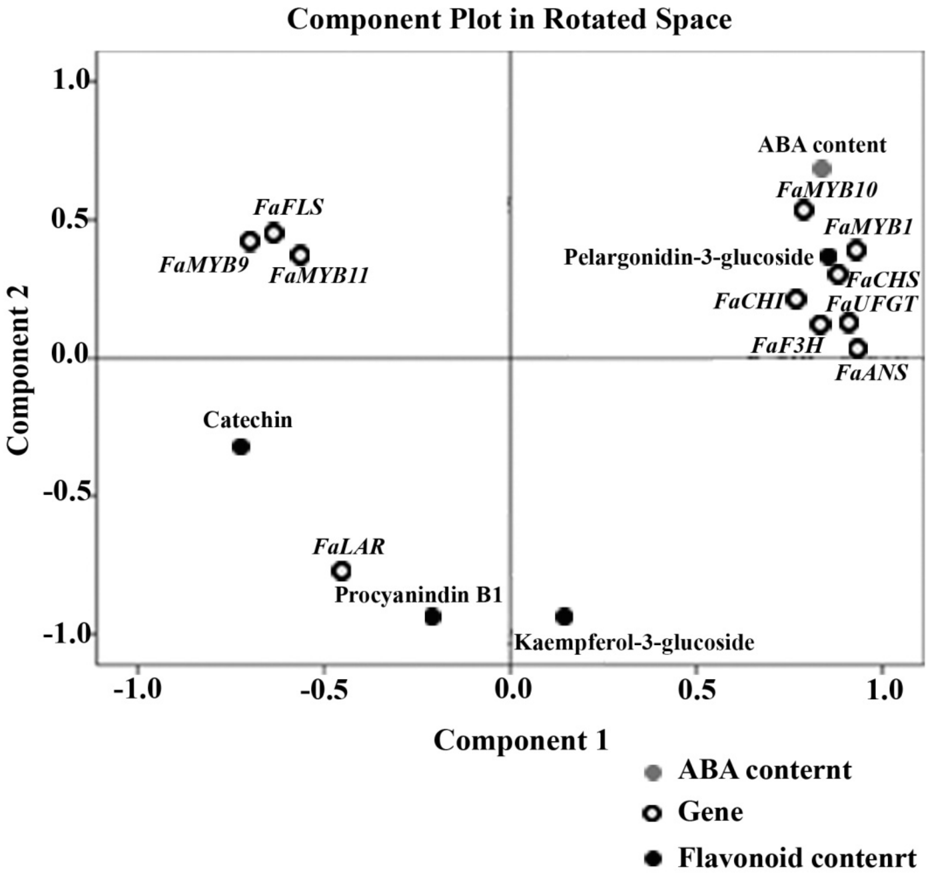

Figure S7 PCA analysis of key flavonoid components against flavonoid biosynthetic structural genes and transcription-related genes. Two PCA components 'anthocyanidin index' and 'proanthocyanin index' explained 80.32% of the variance in fruit coloration of red and white strawberry varieties.

Table S1 Analysis of flavonoid contents.

Flavonoid Components	W-T (μg/g)	R-T (μg/g)	W-R (μg/g)	R-R (μg/g)	W-T/R-T (%)	W-R/R-R (%)
PA	1584.76	2123.50	922.83	1716.64	74.63%	53.76%
AC	189.93	447.65	188.47	126418.85	42.43%	0.15%
FL	25.47	260.28	13.50	52.29	9.79%	25.82%
PP	1623.32	983.71	166.94	209.51	165.02%	79.68%

Note: PA: proanthocyanins, AC: anthocyanins, FL: flavonols, PP: the precursors of proanthocyanidins, catechin, and epi-catechin, W-T: White strawberry turning fruit stage, R-T: Red strawberry turning fruit stage, W-R: White strawberry ripe fruit stage, R-R: Red strawberry ripe stage.

Table S2 The expression of structural genes, *FaANS*, *FaUFGT*, and *FaDFR* in flavonoid biosynthesis in red and white strawberry varieties.

The Measurement of Structural Genes' Transcript Levels	*FaANS*						*FaUFGT*						*FaDFR*					
Developmental stage	G1	G2	W	T	R	OR	G1	G2	W	T	R	OR	G1	G2	W	T	R	OR
Standardized Transcript Level in White Strawberry Variety	1.02 ± 0.13	0.07 ± 0.03	0.05 ± 0.02	0.03 ± 0.02	0.03 ± 0.01	0.10 ± 0.036	1.00 ± 0.03	0.07 ± 0.01	0.12 ± 0.01	0.14 ± 0.01	0.05 ± 0.00	6.94×10^{-3} ± 0.00	1.02 ± 0.12	1.19 ± 0.07	8.59 ± 0.62	7.50 ± 0.48	5.22 ± 0.41	4.70 ± 0.40
Standardized Transcript Level in Red Strawberry Variety	0.47 ± 0.06	0.01± 0.00	7.94×10^{-3} ± 0.00	0.23 ± 0.07	0.81 ± 0.14	0.83 ± 0.19	0.40 ± 0.05	0.13 ± 0.01	2.48 ± 0.38	6.29 ± 0.97	23.16 ± 1.18	38.69 ± 2.97	0.23 ± 0.02	5.16 ± 0.58	8.65 ± 0.65	9.98 ± 1.67	7.88 ± 0.54	10.32 ± 1.20
Transcript Ratio between Red and White Strawberry Variety	0.46	0.15	0.17	6.63	25.23	7.90	0.40	1.87	21.40	44.13	449.61	5572.49	0.23	4.33	1.01	1.33	1.51	2.20

Note: *FaANS*: anthocyanidin synthase; *FaUFGT*: flavonol-O-glucosyltransferases; *FaDFR*: dihydroflavonol-4-reductase.

Table S3 The expression of structural genes, *FaLAR, FaANR,* and *FaFLS* in flavonoid biosynthesis in red and white strawberry varieties.

The Measurement of Structural Genes' Transcript Levels	*FaANR*						*FaLAR*						*FaFLS*					
Developmental Stage	G1	G2	W	T	R	OR	G1	G2	W	T	R	OR	G1	G2	W	T	R	OR
Standardized Transcript Level in White Strawberry Variety	1.00 ± 0.01	0.09 ± 0.01	0.05 ± 0.00	0.03 ± 0.01	1.38×10^{-3} ± 0.00	1.32×10^{-3} ± 0.00	1.00 ± 0.06	0.08 ± 0.01	0.11 ± 0.02	0.04 ± 0.00	6.40×10^{-3} ± 0.00	5.02×10^{-3}± 0.000	1.04 ± 0.20	1.09 ± 0.20	2.83 ± 0.57	1.60 ± 0.24	0.40 ± 0.07	0.07 ± 0.01
Standardized Transcript Level in Red Strawberry Variety	0.17 ± 0.03	4.52×10^{-3} ± 0.00	3.22×10^{-3} ± 0.00	0.08 ± 0.01	0.36 ± 0.10	0.36 ± 0.03	0.21 ± 0.02	0.06 ± 0.01	0.04 ± 0.00	0.06 ± 0.00	0.02 ± 0.00	0.02 ± 0.00	0.71 ± 0.09	5.16 ± 0.83	0.74 ± 0.11	0.10 ± 0.02	0.24 ± 0.04	0.08 ± 0.02
Transcript Ratio between Red and White Strawberry Variety	0.16	0.05	0.07	2.32	259.43	274.45	0.21	0.75	0.39	1.41	2.47	3.41	0.69	4.73	0.26	0.06	0.60	1.22

Note: *FaANR*: anthocyanidin reductase; *FaLAR*: leucoanthocyanidin reductase; *FaFLS*: flavonol synthase.

Table S4 The expression of regulatory genes, *FaMYB1*, *FaMYB10*, and *FaWD40* of flavonoid biosynthesis in red and white strawberry varieties.

The Measurement of Regulatory Genes' Transcript Level	*FaMYB1*						*FaMYB10*						*FaWD40*					
Developmental Stage	G1	G2	W	T	R	OR	G1	G2	W	T	R	OR	G1	G2	W	T	R	OR
Standardized Transcript Level in White Strawberry Variety	1.00 ± 0.06	0.78 ± 0.09	0.69 ± 0.10	0.25 ± 0.01	3.27 ± 0.18	1.53 ± 0.13	1.01 ± 0.11	4.33 ± 0.85	178.42 ± 33.92	120.57 ± 18.38	18245.68 ± 3809.03	7039.18 ± 1502.67	1.01 ± 0.11	2.93 ± 0.86	3.64 ± 0.78	1.56 ± 0.31	1.37 ± 0.31	1.31 ± 0.32
Standardized Transcript Level in Red Strawberry Variety	3.60 ± 0.52	3.28 ± 0.41	0.90 ± 0.06	1.62 ± 0.15	5.39 ± 0.55	7.71 ± 0.99	24.65 ± 9.77	62.78 ± 27.56	697.32 ± 21.21	5408.22 ± 487.18	22578.06 ± 1291.22	22472.76 ± 2072.94	2.74 ± 0.40	4.73 ± 0.79	1.49 ± 0.11	1.35 ± 0.08	1.33 ± 0.47	1.27 ± 0.23
Transcript Ratio between Red and White Strawberry Variety	3.58	4.22	1.32	6.57	1.64	5.03	24.39	14.50	3.91	44.86	1.24	3.19	2.70	1.61	0.41	0.86	0.98	0.97

Table S5 The expression of regulatory genes, *FaMYB9*, and *FaMYB11* of flavonoid biosynthesis in red and white strawberry varieties.

The Measurement of Regulatory Genes' Transcript Level	*FaMYB9*						*FaMYB11*					
Developmental Stage	G1	G2	W	T	R	OR	G1	G2	W	T	R	OR
Standardized Transcript Level in White Strawberry Variety	1.00 ± 0.08	0.46 ± 0.07	0.51 ± 0.07	0.27 ± 0.04	0.07 ± 0.01	0.01 ± 0.00	1.03 ± 0.17	0.41 ± 0.11	0.94 ± 0.21	0.56 ± 0.17	0.08 ± 0.02	0.01 ± 0.00
Standardized Transcript Level in Red Strawberry Variety	1.02 ± 0.11	0.21 ± 0.03	0.02 ± 0.00	0.01 ± 0.00	0.03 ± 0.00	7.37×10^{-3} ± 0.00	3.11 ± 0.48	0.36 ± 0.07	0.06 ± 0.02	0.01 ± 0.00	0.05± 0.00	0.01± 0.00
Transcript Ratio between Red and White Strawberry Variety	1.01	0.45	0.04	0.05	0.40	0.53	3.01	0.88	0.06	0.02	0.60	1.39

Table S6 The sequences of the oligonucleotide primers used in this work.

Gene	Accession	PrimerSequence (5'→3')	Protein	Function	Purpose	Reference (s)
FaMYB10	MG456859	F:ATGGAGGGTTTCGGTGTGAGAAAAGGT GCATGGACTAAAGAGGAAGATGAACTTCTG<br>R: TCATACGTAGGAGATGTTGA	Myb domain protein 10	Regulating anthocyanin biosynthesis	Cloning	Our results
FaMYB1	MG456857	F: ATGAGGAAGCCCTGCTGCGA<br>R: TTAAGCAACTTGAGGATCAG	Myb domain protein 1	Regulating anthocyanin biosynthesis	Cloning	Our results
Actin	AB116565	F: GGGTTTGCTGGAGATGAT<br>R: CATCCCAGTTGCTCACAATA	Actin	Actin	qRT-PCR	Our results
FaMYB1	AF401220	F: CCTGCTGCGAGAAGACGGAGAC<br>R: CTCTTACCACAACGACGCAACCCT	Myb domain protein 1	Regulating anthocyanin biosynthesis	qRT-PCR	Aharoni *et al.*, 2001
FaMYB10	EU155162	F: TCAAATCAGGCTTAAACAGA<br>R: TTAAAGACCACCTGTTTCCT	Myb domain protein 10	Regulating anthocyanin biosynthesis	qRT-PCR	Lin-Wang *et al.*, 2010
FaMYB9	JQ989281	F: CCGGATCATCCAAGGAAACA<br>R: CCTAGTGGCCTTGGTTCTTATT	Myb domain protein 9	Modifying PA content	qRT-PCR	Schaart *et al.*, 2013
FaMYB11	JQ989282	F: GATGGTCTTTGATAGCGGGTAG<br>R: TGGTGGTTTGTGGTGAGTAAT	Myb domain protein 11	Modifying PA content	qRT-PCR	Schaart *et al.*, 2013
FabHLH3-delta	JQ989285	F: ACCGAGTAGTAGCAGACTCCGTGGTAT<br>R: CCATCTGCCCATATTAACATCCCTTG	Basic helix-loop- helix protein	Regulating anthocyanin biosynthesis	qRT-PCR	Schaart *et al.*, 2013
FaWD40	JQ989287	F: GACTTGAGGTACATGGCGACGATTT<br>R: TCATCCCCACCCGAGCAAATAT	W(Trp)D(Asp)-repeat protein	Regulating anthocyanin biosynthesis	qRT-PCR	Schaart *et al.*, 2013

FaPAL	HM641823	F: TGCTGTTGGGTCTGGTATGG<br>R: ACCTTCTTCGCTTCTTTCAC	Phenylalanine ammonia-lyase	Structural genes in flavonoid biosynthesis	qRT-PCR	Pombo *et al.*, 2011
FaC4H	DQ898278	F: TTGCGCAGAGTTTTGAGTATAATT<br>R: TCCTTCATTAGTTGTGACCTGTGT	Cinnamate 4-hydroxylase	Structural genes in flavonoid biosynthesis	qRT-PCR	Saud, *et al.*, 2009
Fa4CL	XM_004309901	F: AAACTCGGACAGGGTTACGG<br>R: CAGTCGCCTCAGGGTCATTC	4-coumarate--CoA ligase	Structural genes in flavonoid biosynthesis	qRT-PCR	Saud, *et al.*, 2009
FaCHS	AY997297	F: CACCTCCTCAAAGATGTTCCTG<br>R: TGCCTCGTGGCTTCTAACTTCT	Chalcone synthase	Structural genes in flavonoid biosynthesis	qRT-PCR	Kadomura-Ishikawa, *et al.* 2015
FaCHI	AB201755	F: TTTCCTCCCTCCGTCAAGCC<br>R: ACTCAACCGACTCTGTCAACTCCT	Chalcone isomerase	Structural genes in flavonoid biosynthesis	qRT-PCR	Kadomura-Ishikawa, *et al.* 2015
FaF3H	AY691918	F: AGGGTGGCTTCATCGTTTCC<br>R: TCAGCTCGTCACTGTACTGCGTTG	Flavanone 3-hydroxylase	Structural genes in flavonoid biosynthesis	qRT-PCR	Almeida *et al.* 2007
FaF3'H	AB665441	F: AAATCAACGGCTACCACATCC<br>R: CCAGCACCAAAGGGTATGAC	Flavonoid 3'-hydroxylase	Structural genes in flavonoid biosynthesis	qRT-PCR	Kadomura-Ishikawa, *et al.* 2015
FaFLS	DQ087252	F: GGCATGTACCAAATCGTGAA<br>R: ATCCACCCACCCTTTCTTCC	Flavonol synthase	Structural genes in flavonoid biosynthesis	qRT-PCR	Almeida *et al.* 2007
FaGT3	AY663786	F: CTGGTGAGTGCGAAGGAAAT<br>R: AAGGAAGTGTATGACGAGCC	UDP-glucose glucosyltransfe	Structural genes in flavonoid	qRT-PCR	Lunkenbein *et al.* 2006

			rase	biosynthesis		
FaDFR	AF029685	F: AAGACAGTTCGGAGGTTGGT<br>R: AGAGTTGGGATAATCGTAATG	Dihydroflavonol 4-reductase	Structural genes in flavonoid biosynthesis	qRT-PCR	Moyano *et al.*, 1998
FaANS	AY695817	F: GAAGTGCGTACCCAACTCCATCGT<br>R: ACCTTCTCCTTGTTGACGAGCCC	Anthocyanidin synthase	Structural genes in flavonoid biosynthesis	qRT-PCR	Almeida *et al.*, 2007
FaUFGT	AY575056	F: TAGCCTTTCCCTTCTCCACTCA<br>R: GTAACGCAGTACGGACGTGTCG	UDP glucose:flavonoid-3-O-glucosyl transferase	Structural genes in flavonoid biosynthesis	qRT-PCR	Griesser *et al.*, 2008
FaANR	JX271492	F: ATGCAAATGCTATCAGGTTCC<br>R: ATGGTCTTCGCTTGGATGGA	Anthocyanidin reductase	Structural genes in flavonoid biosynthesis	qRT-PCR	Schaart *et al.*, 2013
FaLAR	DQ087253	F: GTGAGGCGTGCGATTGAGA<br>R: TCGACGAAGTAGGCTTTAACC	Leucoanthocyanidin reductase	Structural genes in flavonoid biosynthesis	qRT-PCR	Schaart *et al.*, 2013
FaMYB10-2-AD-NdeI<br>*FaMYB10-2*-AD-XhoI	MG456860	F: GAAATTCATATGATGGAGGGTTATTTCGGTGT<br>R: GAAATTCTCGAGCTAATTGTAGAGTCTGTGGTGG	Myb domain protein 10	Regulating anthocyanin biosynthesis	Yeast two-hybrid	Our results
FaMYB10-1-AD-NdeI<br>*FaMYB10-1*-AD-XhoI	MG456859	F: GAAATTCATATGATGGAGGGTTATTTCGGTG<br>R: GAAATTCTCGAGTCATACGTAGGAGATGTTGACT	Myb domain protei 10	Regulating anthocyanin biosynthesis	Yeast two-hybrid	Our results
FaMYB10-1-N-AD-XhoI<br>*FaMYB10-1*-C-AD-Nd	MG456859	R: GAAATTCTCGAGTCATAATTGTAGAGTCTGTGGTGG<br>F: GAAATTCATATGATGGAAAATGTAACTGATTGGTG	Myb domain protein 10	Regulating anthocyanin biosynthesis	Yeast two-hybrid	Our results

eI						
FaWD40-AD＆<br>BK-NdeI<br>*FaWD40*-AD＆<br>BK-BamHI	JQ989287	F: GAAATTCATATGATGGAGAATTCGACCCTCGA<br>R: GAAATTGGATCCTCAAACCTTCAAGAGCTGCATC	WD-repeat protein	Regulating anthocyanin biosynthesis	Yeast two-hybrid	Our results
PavbHLH-AD＆<br>BK-EcoRI<br>*PavbHLH*-AD＆<br>BK-PstI	KP126521	F: GAAATTGAATTCATGGCTGCACCGCCAAGCAGCA<br>R: GAAATTCTGCAGTCAGATTGGGGAATTATTTGATTTA	Basic helix-loop- helix protein	Regulating anthocyanin biosynthesis	Yeast two-hybrid	Our results
FaMYB10-HT-AgeI<br>*FaMYB10*-HT-XhoI	MG456859	F: GCGACCGGTATGGAGGGTTTCGGTGTGAGA<br>R:GGCCTCGAGTCATACGTAGGAGATGTTGACTAGATGA	Myb domain protein 10	Regulating anthocyanin biosynthesis	Transient expression	Our results

Note: F, forward; R, reverse. Underlines represent the digestion sites of enzymes.

Table S7 Information about the MYB protein sequences used for the construction of the neighbor joining tree.

Gene	Accession	Protein	Function	Identity	Reference(s)
AtMYB3	NP_564176.2	Myb domain protein 3	Repressors of phenylpropanoid pathway	51% ident with FaMYB1	Lin-Wang *et al.*, 2010
AtMYB4	AAC83582.1	Myb domain protein 4	Repressors of phenylpropanoid pathway	50% ident with FaMYB1	Aharoni *et al.*, 2001
AtMYB6	NP_192684.1	myb domain protein 6	Repressors of phenylpropanoid pathway	53% ident with FaMYB1	Lin-Wang *et al.*, 2010
AtMYB24	NP_198851.1	myb domain protein 24	Anther development	100% ident with AtMYB24	Yang *et al.*, 2007
AtMYB75	AAG42001.1	Myb domain protein 75	Regulating anthocyanin biosynthesis	41% ident with FaMYB10	Gonzalez *et al.*, 2008
AtMYB90	NP_176813.1	Myb domain protein 90	Regulating anthocyanin biosynthesis	42% ident with FaMYB10	Gonzalez *et al.*, 2008
FaMYB1	AAK84064.1	Myb domain protein 1	Regulating anthocyanin biosynthesis by interacting with other anthocyanin regulators	100% ident with FaMYB1	Aharoni *et al.*, 2001
FcMYB1	ADK56163.1	Myb domain protein 1	Regulating branching-point of the anthocyanin/PA biosynthesis	100% ident with FaMYB1	Salvatierra *et al.*, 2013
FaMYB1-1	MG456857	Myb domain protein 1	Regulating anthocyanin biosynthesis	100% ident with FaMYB1	Our results
FaMYB1-2	MG456858	Myb domain protein 1	Regulating anthocyanin biosynthesis	100% ident with FaMYB1	Our results
FaMYB9	AFL02460.1	Myb domain protein 9	Modifying PA content	100% ident with FaMYB9	Schaart *et al.*, 2013
FaMYB10	ABX79947.1	Myb domain protein 10	Regulating anthocyanin biosynthesis	100% ident with FaMYB10	Lin-Wang *et al.*, 2010
FvMYB10	ABX79948.1	Myb domain protein 10	Regulating anthocyanin biosynthesis	93% ident with FaMYB10	Lin-Wang *et al.*, 2010
FaMYB10-1	MG456859	Myb domain protein 10	Regulating anthocyanin biosynthesis	98% ident with FaMYB10	Our results
FaMYB10-2	MG456860	Myb domain protein 10	Regulating anthocyanin biosynthesis	91% ident with FaMYB10	Our results
FaMYB11	AFL02461.1	Myb domain protein1 1	Modifying PA content	100% ident with FaMYB11	Schaart *et al.*, 2013
FvMYB305-like	XP_011468270.1	Myb domain protein 305	Probably anther development	61% ident with AtMYB24	NCBI database
MdMYB1	ABK58136.1	Myb domain protein 1	Regulating anthocyanin biosynthesis	46% ident with FaMYB10	Takos *et al.*, 2006
MdMYB6-like	XP_008374825.1	Myb domain protein 6	Probably regulating anthocyanin biosynthesis	61% ident with FaMYB1	Gao *et al.*, 2011
MdMYB9	NP_001280749.1	Myb domain protein 9	Regulating anthocyanin and PA biosyntheris	66% ident with FaMYB9	An *et al.*, 2015
MdMYB10	ACQ45201.1	Myb domain protein 10	Regulating anthocyanin biosynthesis	46% ident with FaMYB10	Espley *et al.*, 2007

MdMYB11	NP_001280958.1	Myb domain protein 11	Regulating anthocyanin and PA biosyntheris	62% ident with FaMYB11	An *et al.*, 2015
MdMYB16	ADL36756.1	Myb domain protein 16	Inhibiting anthocyanin synthesis	52% ident with FaMYB1	Xu *et al.*, 2017
MdMYB17	ADL36757.1	Myb domain protein 17	Inhibiting anthocyanin synthesis	56% ident with FaMYB1	Lin-Wang *et al.*, 2011
MdMYB21	NP_001280981.1	Myb domain protein 21	Probably anther development	43% ident with AtMYB24	NCBI database
MdMYB111	ADL36754.1	Myb domain protein 111	Inhibiting anthocyanin synthesis	60% ident with FaMYB1	Lin-Wang *et al.*, 2011
MdMYB305-like	XP_008341440.1	Myb domain protein 305	Probably anther development	66% ident with AtMYB24	NCBI database
MsMYB10	ABX71485.1	Myb domain protein 10	Regulating anthocyanin biosynthesis	46% ident with FaMYB10	Wang *et al.*, 2010
ParMYB10	ABX71490.1	Myb domain protein 10	Regulating anthocyanin biosynthesis	51% ident with FaMYB10	Lin-Wang *et al.*, 2010
PavMYB10.1	ALM31951.1	Myb domain protein 10	Regulating anthocyanin biosynthesis	55% ident with FaMYB10	Jin *et al.*, 2016
PavMYB11	ALH21142.1	Myb domain protein 10	Probably regulating PA biosynthesis	61% ident with FaMYB11	NCBI database
PavMYB111	ALH21138.1	Myb domain protein111	Probably regulating anthocyanin biosynthesis	61% ident with FaMYB1	NCBI database
PavMYBR	ADY15315.1	Myb domain protein R	Regulating flavonoid biosynthesis	51% ident with FaMYB1	NCBI database
PcfMYB10	ABX71495.1	Myb domain protein 10	Regulating anthocyanin biosynthesis	52% ident with FaMYB10	Lin-Wang *et al.*, 2010
PdmMYB10	ABX71492.1	Myb domain protein 10	Regulating anthocyanin biosynthesis	50% ident with FaMYB10	Lin-Wang *et al.*, 2010
PpyMYB10	ABX71488.1	Myb domain protein 10	Regulating anthocyanin biosynthesis	47% ident with FaMYB10	Lin-Wang *et al.*, 2010
VvMYBA1	BAE96751.1	Myb domain protein A1	Regulating anthocyanin biosynthesis	44% ident with FaMYB10	Yakushiji *et al.*, 2006
VvMYB6	XP_002273328.1	Myb domain protein 6	Transcription repressor	61% ident with FaMYB1	NCBI database
VvMYB24	NP_001268062.1	Myb domain protein 24	Probably regulating auther develpment	65% ident with AtMYB24	NCBI database
VvMYB308	XP_010648383.1	Myb domain protein 308	Probably regulating PA biosynthesis	57% ident with FaMYB9	NCBI database

References

Aharoni, A., De Vos, C.H.R., Wein, M., Sun, Z.K., Greco, R., Kroon, A., Mol, J.N.M. *et al.* (2001) The strawberry *FaMYB1* transcription factor suppresses anthocyanin and flavonol accumulation in transgenic tobacco. *Plant J.* **28**, 319-332.

Almeida, J.R.M., D'Amico, E., Preuss, A., Carbone, F., de Vos, C.H.R., Deiml, B., Mourgues, F. *et al.* (2007) Characterization of major enzymes and genes involved in flavonoid and proanthocyanidin biosynthesis during fruit development in strawberry (*Fragaria* × *ananassa*). *Arch. Biochem. Biophys.* **465**, 61-71.

An, X.H., Tian, Y., Chen, K.Q., Liu, X.J., Liu, D.D., Xie, X.B., Cheng, C.G. *et al.* (2015) *MdMYB9* and *MdMYB11* are Involved in the regulation of the JA-induced biosynthesis of anthocyanin and proanthocyanidin in apples. *Plant Cell Physiol.* **56**, 650-662.

Espley, R.V., Hellens, R.P., Putterill, J., Stevenson, D.E., Kutty-Amma, S. and Allan, A.C. (2007) Red colouration in apple fruit is due to the activity of the MYB transcription factor, *MdMYB10*. *Plant J.* **49**, 414-427.

Gao, J.J., Shen, X.F., Zhang, Z., Peng, R.H., Xiong, A.S., Xu, J., Zhu, B. *et al.* (2011) The myb transcription factor *MdMYB6* suppresses anthocyanin biosynthesis in transgenic *Arabidopsis*. *Plant Cell Tiss. Org.* **106**, 235-242.

Gonzalez, A., Zhao, M., Leavitt, J.M. and Lloyd, A.M. (2008) Regulation of the anthocyanin biosynthetic pathway by the *TTG1*/*bHLH*/*Myb* transcriptional complex in *Arabidopsis* seedlings. *Plant J.* **53**, 814-827.

Griesser, M., Hoffmann, T., Bellido, M.L., Rosati, C., Fink, B., Kurtzer, R., Aharoni, A. *et al.* (2008) Redirection of flavonoid biosynthesis through the down-regulation of an anthocyanidin glucosyltransferase in ripening strawberry fruit. *Plant Physiol.* **146**, 1528-1539.

Jin, W.M., Wang, H., Li, M.F., Wang, J., Yang, Y., Zhang, X.M., Yan, G.H. *et al.* (2016) The R2R3 MYB transcription factor *PavMYB10.1* involves in anthocyanin biosynthesis and determines fruit skin colour in sweet cherry (*Prunus avium* L.). *Plant Biotechnol. J.* **14**, 2120-2133.

Kadomura-Ishikawa, Y., Miyawaki, K., Takahashi, A., Masuda, T. and Noji, S. (2015) Light and abscisic acid independently regulated *FaMYB10* in *Fragaria* x *ananassa* fruit. *Planta* **241**, 953-965.

Lin-Wang, K., Bolitho, K., Grafton, K., Kortstee, A., Karunairetnam, S., McGhie, T., Espley, R. *et al.* (2010) An R2R3 *MYB* transcription factor associated with regulation of the anthocyanin biosynthetic pathway in Rosaceae. *BMC Plant Biol.* **10**, 50.

Lin-Wang, K.U.I., Micheletti, D., Palmer, J., Volz, R., Lozano, L., Espley, R., Hellens, R.P. *et al.* (2011) High temperature reduces apple fruit colour via modulation of the anthocyanin regulatory complex. *Plant Cell Environ.* **34**, 1176-1190.

Lunkenbein, S., Bellido, M., Aharoni, A., Salentijn, E.M.J., Kaldenhoff, R., Coiner, H.A., Muñoz-Blanco, J. *et al.* (2006) Cinnamate metabolism in ripening fruit. characterization of a UDP-Glucose:cinnamate glucosyltransferase from strawberry. *Plant Physiol.* **140**, 1047-1058.

Moyano, E., Portero-Robles, I., Medina-Escobar, N., Valpuesta, V., Muñoz-Blanco, J. and Luis Caballero, J. (1998) A fruit-specific putative dihydroflavonol 4-reductase gene is differentially expressed in strawberry during the ripening process. *Plant Physiol.* **117**, 711-716.

Pombo, M., Martínez, G. and Civello, P. (2011) Cloning of FaPAL6 gene from strawberry fruit and characterization of its expression and enzymatic activity in two cultivars with different anthocyanin accumulation. *Plant Sci.* **181**, 111-118.

Salvatierra, A., Pimentel, P., Moya-León, M.A. and Herrera, R. (2013) Increased accumulation of

anthocyanins in *Fragaria chiloensis* fruits by transient suppression of *FcMYB1* gene. *Phytochemistry* **90**, 25-36.

Saud, G., Carbone, F., Perrotta, G., Figueroa, C.R., Moya, M., Herrera, R., Retamales, J.B. *et al.* (2009) Transcript profiling suggests transcriptional repression of the flavonoid pathway in the white-fruited Chilean strawberry, *Fragaria chiloensis* (L.) Mill. *Genet. Resour. Crop Ev.* **56**, 895–903.

Schaart, J.G., Dubos, C., De La Fuente, I.R., van Houwelingen, A.M.M.L., de Vos, R.C.H., Jonker, H.H., Xu, W.J. *et al.* (2013) Identification and characterization of MYB-bHLH-WD40 regulatory complexes controlling proanthocyanidin biosynthesis in strawberry (*Fragaria* x *ananassa*) fruits. *New Phytol.* **197**, 454-467.

Takos, A.M., Jaffe, F.W., Jacob, S.R., Bogs, J., Robinson, S.P. and Walker, A.R. (2006) Light-induced expression of a *MYB* gene regulates anthocyanin biosynthesis in red apples. *Plant Physiol.* **142**, 1216-1232.

Wang, Y., Zhang, Y., Feng, S., Tian, C., Wang, H., Liu, Z., Song, Y. and Chen, X. (2010) Cloning, sequence analysis and expression in *E. coli* of MsMYB10 gene from *Malus sieversii* f. *neidzwetzkyana*. *Scientia Agricultura Sinica* **43**, 2735-2743.

Xu, H., Wang, N., Liu, J., Qu, C., Wang, Y., Jiang, S., Lu, N. *et al.* (2017) The molecular mechanism underlying anthocyanin metabolism in apple using the MdMYB16 and MdbHLH33 genes. *Plant Mol. Biol.* **94**, 149-165.

Yakushiji, H., Kobayashi, S., Goto-Yamamoto, N., Tae Jeong, S., Sueta, T., Mitani, N. and Azuma, A. (2006) A skin color mutation of grapevine, from black-skinned pinot noir to white-skinned pinot blanc, is caused by deletion of the functional *VvmybA1* allele. *Biosci. Biotech. Bioch.***70**, 1506-1508.

Yang, X., Li, J., Pei, M., Gu, H., Chen, Z. and Qu, L. J. (2007) Over-expression of a flower-specific transcription factor gene *AtMYB24* causes aberrant anther development. *Plant Cell Rep.* **26**, 219-228.
